# Real-world safety of carboplatin in non-small cell lung cancer: a retrospective signal detection and subgroup analysis based on the FAERS database

**DOI:** 10.3389/fmed.2025.1590738

**Published:** 2025-06-16

**Authors:** Lei Wang, Kunpeng Yang, Hui Zhao, Peiyun Lv, Chenglun Cai, Zhe Wang, Bao Wang

**Affiliations:** ^1^Jilin Cancer Hospital, Changchun, China; ^2^Changchun University of Chinese Medicine, Changchun, China; ^3^Tianjin Medical University, Tianjin, China

**Keywords:** carboplatin, NSCLC, adverse events, pharmacovigilance, subgroup analysis

## Abstract

**Introduction:**

Carboplatin is frequently employed in the treatment of non-small cell lung cancer (NSCLC), yet the real-world safety profile—including underrecognized adverse events (AEs) and subgroup-specific risk variations—remains incompletely understood. This study aims to systematically assess carboplatin-related AEs and explore demographic factors that may influence risk.

**Methods:**

A retrospective analysis was performed using data from the FDA Adverse Event Reporting System (FAERS) spanning the first quarter of 2004 to the third quarter of 2024. Standardized terminology harmonization and multiple disproportionality methods—including reporting odds ratio (ROR), proportional reporting ratio (PRR), and Bayesian analysis—were applied to detect potential safety signals. Subgroup analyses were conducted to identify sex- and age-specific variations in risk.

**Results:**

Among 4,748 reports meeting inclusion criteria, known hematologic toxicities (e.g., anemia, neutropenia) and renal impairment were confirmed. Additionally, previously unlabeled risks emerged, such as abdominal pain (higher incidence in females), neutropenic sepsis (predominant in males and older adults), and hypothyroidism. Subgroup analyses revealed distinct patterns: males exhibited increased infection-related events, whereas females were more prone to gastrointestinal and hepatic complications. Patients aged ≥65 years showed increased multisystem involvement, particularly affecting hematologic and renal functions.

**Discussion:**

These findings underscore the necessity of tailored monitoring strategies for carboplatin, taking into account patient sex and age, especially when used in conjunction with immunotherapy. The study’s insights support refining pharmacovigilance strategies and updating clinical guidelines to enable early intervention and improve personalized management for patients with NSCLC.

## 1 Introduction

Lung cancer remains one of the leading causes of cancer-related mortality worldwide, with non-small cell lung cancer (NSCLC) accounting for approximately 85% of all cases (1, 2). Recent advances in genetic testing and molecular profiling have guided the development of personalized targeted therapies, including those aimed at EGFR, ALK, and ROS1 mutations. These targeted approaches have significantly improved the prognosis for certain patients with advanced NSCLC (3, 4). Likewise, the advent of immune checkpoint inhibitors has transformed the treatment landscape for metastatic and locally advanced NSCLC, enabling some patients to achieve long-term survival (5–8). Nevertheless, traditional cytotoxic chemotherapy remains a cornerstone of NSCLC management, especially for those patients who are ineligible for or have not yet received immunotherapy or targeted treatments. Platinum-based combination regimens thus continue to play a critical role in these circumstances (8–10).

Carboplatin, a second-generation platinum-based agent, offers better tolerability than cisplatin, particularly with respect to nephrotoxicity and neurotoxicity (11, 12). Consequently, carboplatin is widely used in NSCLC treatment, especially among patients unable to tolerate cisplatin or presenting with multiple comorbidities (9, 13). Although most previous studies have centered on carboplatin’s therapeutic efficacy, systematic analyses of rare or severe adverse events (AEs) in large populations remain relatively scarce (10, 14). In clinical practice, carboplatin-induced hematological toxicities—including neutropenia, thrombocytopenia, and anemia—alongside renal impairment and other complications, continue to be major concerns (11, 15). Moreover, the safety profile of carboplatin used in combination with immune checkpoint inhibitors or other agents still requires clarification. Debates also persist over optimal dosing strategies, particularly regarding accurate renal function assessments and standardized area under the curve (AUC)-based dosing methods (12, 16). Collectively, these concerns underscore the need for comprehensive evaluations of carboplatin-related adverse events in large-scale datasets.

Despite carboplatin’s extensive use, real-world safety assessments of this agent in NSCLC remain limited. Several gaps persist in the current literature. First, large randomized controlled trials (RCTs) typically implement strict inclusion criteria for patient demographics and treatment regimens, restricting their ability to capture the full heterogeneity of real-world patients, including older adults, those with multiple comorbidities, or patients from diverse ethnic backgrounds. As a result, rare or delayed-onset adverse events may be underestimated (6, 7, 10, 17). Second, existing carboplatin safety studies have primarily addressed common hematological toxicities, with insufficient attention to potential risks in other organ systems—such as cardiovascular, neurological, hepatic, or renal—that may arise from monotherapy or combination regimens (8, 13, 14, 18). Third, as immunotherapy and other novel modalities gain prominence, carboplatin is often incorporated into combination regimens. This emerging treatment context may shift carboplatin’s adverse event profile, warranting further study through large-scale safety monitoring databases (3, 5, 19). Finally, in the arena of real-world evidence (RWE), systematic detection, extraction, and analysis of serious adverse events related to carboplatin using the FDA Adverse Event Reporting System (FAERS) database remain underexplored, signifying a need for refined methodologies (20).

To fill these knowledge gaps, the present study systematically evaluates the real-world safety profile of carboplatin in NSCLC patients. Specifically, we obtained carboplatin-related adverse event reports from the FAERS database spanning the first quarter of 2004 to the third quarter of 2024. Through de-duplication, standardized adverse event terminology, and multiple signal detection approaches—namely, reporting odds ratio (ROR), proportional reporting ratio (PRR), and Bayesian analysis—we assessed major adverse events and newly emerging signals linked to carboplatin. Accordingly, this research addresses two core questions: (1) Does real-world carboplatin use reveal any serious safety signals underrecognized or underemphasized in existing literature and labeling? (2) How do patterns and distributions of adverse events differ among various subgroups, such as sex, age, or concurrent medication use?

The importance of this study is twofold. From a clinical standpoint, carboplatin sees extensive use across different subgroups of NSCLC patients, yet most safety data derives from small-scale or highly selected populations. Utilizing FAERS’s large sample size and near–real-time monitoring capabilities, our research offers clinicians and pharmacists more accurate risk assessments, enabling enhanced peri-treatment monitoring and intervention strategies. From a pharmacovigilance and health policy perspective, reappraising carboplatin’s safety via FAERS—a major global spontaneous reporting system—may assist regulatory bodies, hospital pharmacy departments, and researchers in detecting new safety signals, optimizing clinical guidelines, and improving treatment recommendations. In addition, the methodological framework employed in this study could serve as a model for investigating the real-world safety of other oncology agents, providing broad applicability and practical guidance (19).

## 2 Materials and methods

### 2.1 Data sources, management process, and study design overview

This study was conducted using the FDA Adverse Event Reporting System (FAERS) database, which compiles spontaneous reports from various sources, including consumers (CN), pharmacists (PH), physicians (MD), other healthcare professionals (HP), and registered nurses (RN). The study period extended from the first quarter of 2004 to the third quarter of 2024, and encompassed all adverse event reports in which carboplatin was designated as the primary suspect.

**Figure 1:**
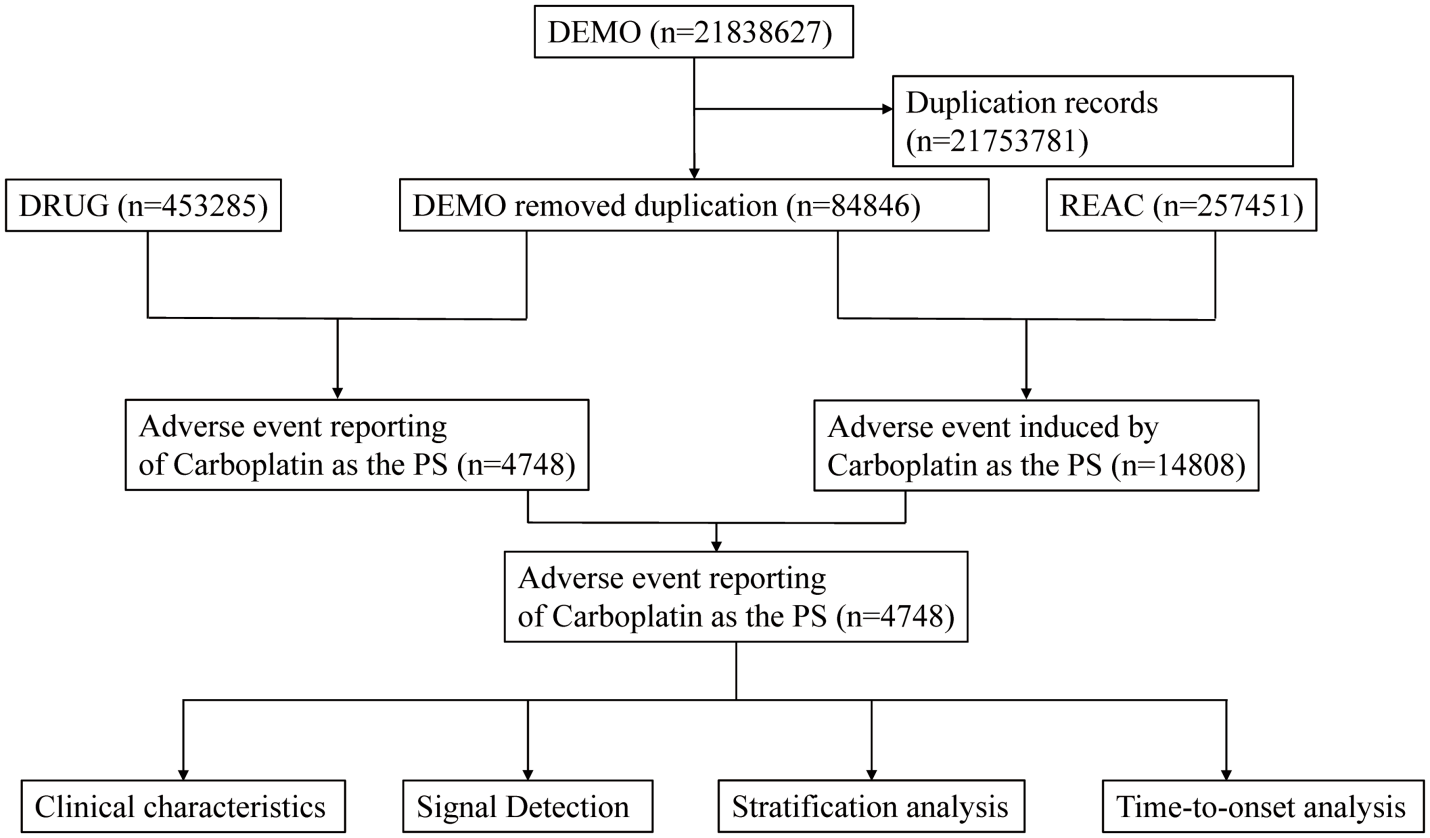
Flowchart demonstrating the adverse event analysis process for Carboplatin using the FDA Adverse Event Reporting System (FAERS) database.

Data management procedures adhered to FDA-recommended standard operating protocols and involved two major steps: removing duplicate reports and standardizing adverse event terms. Duplicate reports were identified and discarded as follows: if multiple reports shared the same CASEID, only the record with the most recent FDA receive date (FDA_DT) was kept; if the CASEID and FDA_DT were both identical, the report with the highest PRIMARYID was retained. Adverse event terms were standardized using version 26.1 of the MedDRA dictionary to ensure consistency in subsequent analyses. A detailed overview of the study workflow is presented in Figure 1.

### 2.2 Statistical analysis methods

A comprehensive descriptive analysis of carboplatin-related adverse event reports was performed initially. Four disproportionality analysis techniques were then applied to probe potential adverse reactions: Reporting Odds Ratio (ROR), Proportional Reporting Ratio (PRR), the Multi-Item Gamma Poisson Shrinker (MGPS), and the Bayesian Confidence Propagation Neural Network (BCPNN). An adverse event qualified as a potential adverse reaction if it surpassed the positive threshold in at least one of these methods. Detailed evaluation criteria and thresholds for each approach are available in Supplementary Tables S1, S2.

The occurrence time of an adverse event was defined as the interval from the initiation of carboplatin therapy (recorded in the THER file) to the adverse event onset (recorded in the DEMO file). A Weibull distribution was applied to model and evaluate temporal trends in adverse event incidence. All statistical analyses were performed using R software (version 4.4.0).

## 3 Results

### 3.1 Descriptive analysis

A total of 4,748 adverse event (AE) reports were included in this study, encompassing 14,808 individual AEs, all of which designated carboplatin as the primary suspect drug. Among these patients, 2,212 were male (46.6%) and 1,451 were female (30.6%), with the majority (39.3%) aged between 65 and 85 years. Healthcare professionals submitted 90.7% of the reports. Regarding geographic distribution, the United States contributed 23.3% of the reports, followed by Germany (21.8%), Japan (9.1%), the United Kingdom (7.0%), and France (6.3%). Further descriptive details are listed in Table 1.

**Table 1:** Clinical characteristics of Carboplatin adverse event reports from the FAERS database (Q1 2004 - Q3 2024).

Characteristics	Case numbers	Case proportion (%)
Number of events	4,748	
Gender		
Male	2,212	46.6
Female	1,451	30.6
Miss	1,085	22.9
Age		
Median (IQR)	66 (58, 76)	
<18	6	0.1
18–64.9	1,457	30.7
65–84.9	1865	39.3
>85	9	0.2
Miss	1,411	29.7
Top 5 reported countries		
United States	1,107	23.3
Germany	1,035	21.8
Japan	433	9.1
United Kingdom	333	7.0
France	297	6.3
Reporter		
Healthcare professional	4,307	90.7
Non-healthcare professional	342	7.2
Miss	99	2.1
Top5 reporting years		
2018	592	12.5
2024	551	11.6
2022	458	9.7
2023	455	9.6
2020	436	9.2

IQR, interquartile range.

### 3.2 Distribution of adverse events at the system organ class (SOC) level

**Figure 2:**
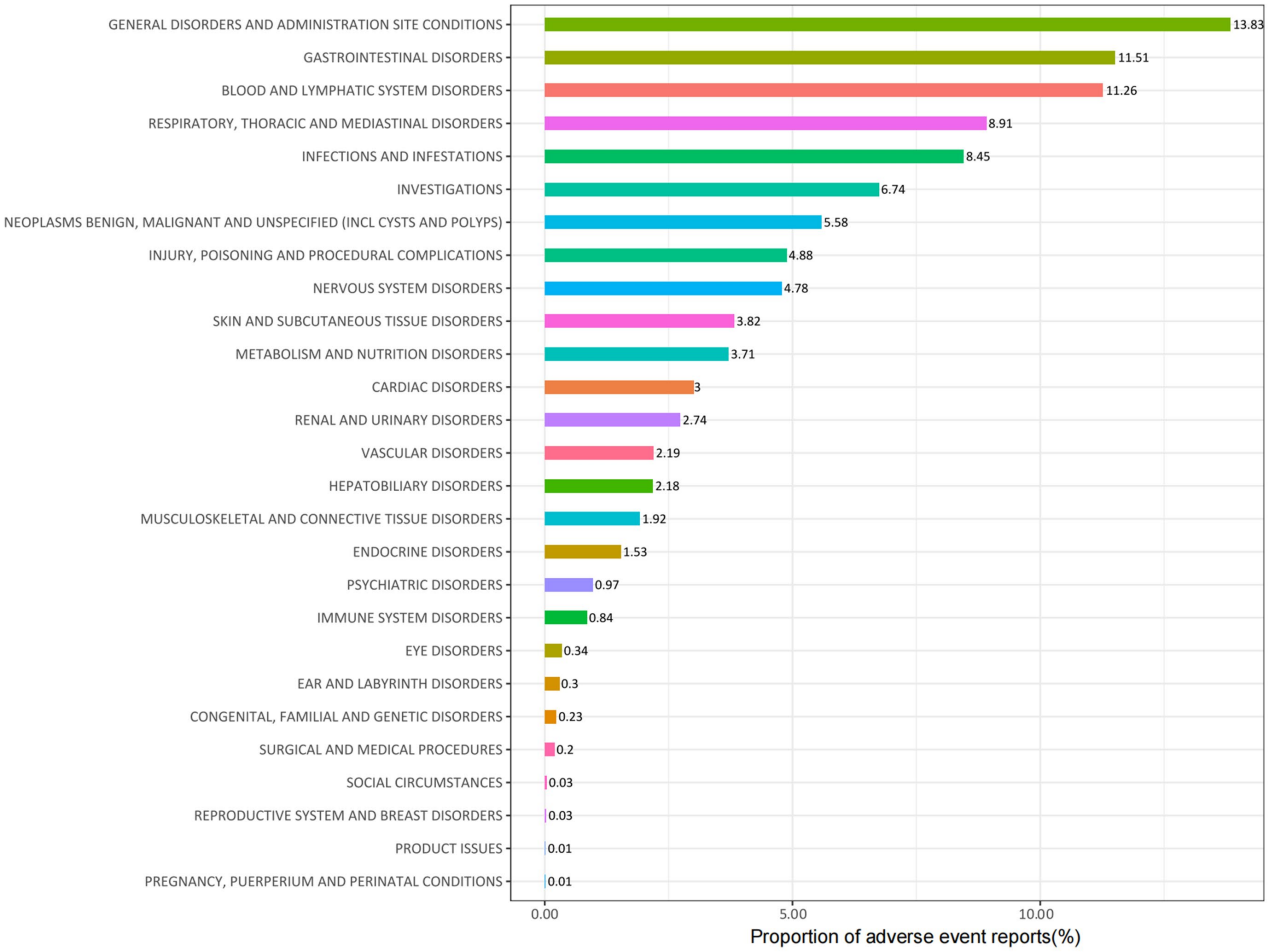
Proportion of adverse events by system organ class associated with Carboplatin.

Carboplatin-related adverse events were classified into 27 SOC categories. As shown in Table 2, key findings emerged in several categories, including INJURY, POISONING AND PROCEDURAL COMPLICATIONS, BLOOD AND LYMPHATIC SYSTEM DISORDERS, INFECTIONS AND INFESTATIONS, RENAL AND URINARY DISORDERS, IMMUNE SYSTEM DISORDERS, and ENDOCRINE DISORDERS. Figure 2 presents the distribution of these adverse events across the respective SOC levels.

**Table 2:** Signal strength of Carboplatin AEs across System Organ Classes (SOC) in the FAERS database.

System organ class (SOC)	Case numbers	ROR(95%Cl)	PRR(χ2)	EBGM(EBGM05)	IC(IC025)
General disorders and administration site conditions	2,048	0.96 (0.92–1.01)	0.97 (2.12)	0.97 (0.93)	−0.04 (−0.11)
Neoplasms benign, malignant and unspecified (incl cysts and polyps)	827	0.67 (0.62–0.72)	0.69 (121.36)	0.7 (0.66)	−0.51 (−0.62)
Injury, poisoning and procedural complications*	722	1.31 (1.21–1.42)	1.29 (46.6)	1.27 (1.19)	0.35 (0.23)
Blood and lymphatic system disorders*	1,668	2.59 (2.45–2.73)	2.41 (1261.53)	2.23 (2.13)	1.16 (1.08)
Metabolism and nutrition disorders	549	0.93 (0.85–1.01)	0.93 (2.89)	0.93 (0.87)	−0.1 (−0.23)
Respiratory, thoracic and mediastinal disorders	1,319	0.84 (0.8–0.89)	0.86 (32.82)	0.87 (0.82)	−0.21 (−0.29)
Infections and infestations*	1,252	1.36 (1.28–1.45)	1.33 (102.94)	1.31 (1.24)	0.39 (0.3)
Gastrointestinal disorders	1,704	1.06 (1–1.11)	1.05 (4.19)	1.05 (1)	0.07 (−0.01)
Renal and urinary disorders*	405	1.2 (1.09–1.33)	1.2 (12.53)	1.18 (1.09)	0.24 (0.09)
Nervous system disorders	708	0.88 (0.82–0.96)	0.89 (9.71)	0.9 (0.84)	−0.16 (−0.27)
Cardiac disorders	444	0.93 (0.84–1.02)	0.93 (2.23)	0.93 (0.86)	−0.1 (−0.24)
Investigations	998	0.84 (0.79–0.9)	0.85 (27.26)	0.86 (0.81)	−0.22 (−0.32)
Musculoskeletal and connective tissue disorders	284	0.67 (0.59–0.75)	0.67 (44.11)	0.69 (0.62)	−0.54 (−0.72)
Vascular disorders	325	0.97 (0.87–1.09)	0.97 (0.23)	0.97 (0.89)	−0.04 (−0.2)
Skin and subcutaneous tissue disorders	566	0.67 (0.62–0.73)	0.69 (83.08)	0.7 (0.65)	−0.52 (−0.64)
Immune system disorders*	125	1.37 (1.14–1.64)	1.36 (11.28)	1.34 (1.15)	0.42 (0.15)
Psychiatric disorders	143	0.65 (0.55–0.76)	0.65 (26.5)	0.66 (0.58)	−0.59 (−0.84)
Hepatobiliary disorders	323	0.94 (0.84–1.06)	0.95 (0.97)	0.95 (0.86)	−0.08 (−0.24)
Surgical and medical procedures	30	0.58 (0.41–0.84)	0.58 (8.56)	0.6 (0.44)	−0.74 (−1.27)
Congenital, familial and genetic disorders	34	0.97 (0.68–1.37)	0.97 (0.04)	0.97 (0.73)	−0.05 (−0.55)
Reproductive system and breast disorders	4	0.16 (0.06–0.43)	0.16 (17.26)	0.17 (0.07)	−2.56 (−3.86)
Endocrine disorders*	227	1.25 (1.09–1.43)	1.25 (10.47)	1.23 (1.1)	0.3 (0.1)
Ear and labyrinth disorders	44	1.13 (0.83–1.54)	1.13 (0.65)	1.12 (0.87)	0.17 (−0.28)
Eye disorders	50	0.27 (0.21–0.36)	0.28 (93.8)	0.29 (0.23)	−1.79 (−2.2)
Social circumstances	5	0.38 (0.16–0.92)	0.38 (4.94)	0.39 (0.19)	−1.34 (−2.54)
Product issues	2	0.27 (0.07–1.11)	0.27 (3.76)	0.29 (0.09)	−1.8 (−3.49)
Pregnancy, puerperium and perinatal conditions	2	1.31 (0.31–5.53)	1.31 (0.13)	1.29 (0.39)	0.36 (−1.39)

Asterisks (*) indicate statistically significant signals in algorithm; ROR, reporting odds ratio; PRR, proportional reporting ratio; EBGM, empirical Bayesian geometric mean; EBGM05, the lower limit of the 95% CI of EBGM; IC, information component; IC025, the lower limit of the 95% CI of the IC; CI, confidence interval; AEs, adverse events.

### 3.3 Distribution of adverse events at the preferred term (PT) level

A frequency ranking and signal assessment were performed for carboplatin-related adverse events. Among the top 50 most frequently reported events, recognized reactions included ANAEMIA, NAUSEA, FATIGUE, THROMBOCYTOPENIA, NEUTROPENIA, FEBRILE NEUTROPENIA, LEUKOPENIA, GENERAL PHYSICAL HEALTH DETERIORATION, PANCYTOPENIA, INFECTION, PLATELET COUNT DECREASED, MUCOSAL INFLAMMATION, ALOPECIA, WHITE BLOOD CELL COUNT DECREASED, HYPOTENSION, HYPOKALAEMIA, POLYNEUROPATHY, NEUROPATHY PERIPHERAL, and HAEMOGLOBIN DECREASED. Additionally, several potential adverse reactions not listed on current product labeling were detected, such as PNEUMONIA, DEHYDRATION, ACUTE KIDNEY INJURY, CONSTIPATION, SEPSIS, PULMONARY EMBOLISM, ABDOMINAL PAIN, HEPATITIS, NEUTROPENIC SEPSIS, DYSPHAGIA, CHEST PAIN, and HYPOTHYROIDISM. A comprehensive summary is provided in Table 3, and all positively signaled events appear in Supplementary Table S3.

**Table 3:** Top 50 frequency of adverse events at the PT level for Carboplatin in FAERS.

PT	Case numbers	ROR(95%Cl)	PRR(χ2)	EBGM(EBGM05)	IC(IC025)
Anaemia*	367	2.41 (2.15–2.69)	2.37 (256.96)	2.2 (2)	1.14 (0.97)
Diarrhoea	290	0.82 (0.73–0.93)	0.82 (10.52)	0.83 (0.75)	−0.26 (−0.44)
Pneumonia*	279	1.35 (1.2–1.53)	1.35 (23.23)	1.32 (1.19)	0.4 (0.22)
Nausea*	277	1.28 (1.13–1.45)	1.28 (15.56)	1.26 (1.13)	0.33 (0.15)
Fatigue*	246	1.26 (1.11–1.44)	1.26 (12.17)	1.24 (1.11)	0.31 (0.12)
Thrombocytopenia*	240	2.63 (2.3–3.02)	2.61 (206.68)	2.39 (2.13)	1.26 (1.06)
Neutropenia*	224	2.19 (1.9–2.52)	2.17 (125.77)	2.03 (1.81)	1.02 (0.82)
Febrile neutropenia*	210	2.26 (1.96–2.62)	2.25 (128.57)	2.1 (1.86)	1.07 (0.86)
Dyspnoea	200	0.94 (0.82–1.09)	0.94 (0.67)	0.95 (0.84)	−0.08 (−0.29)
Leukopenia*	194	4.5 (3.83–5.27)	4.45 (409.42)	3.71 (3.25)	1.89 (1.66)
Vomiting	184	1.14 (0.98–1.32)	1.13 (2.74)	1.12 (0.99)	0.17 (−0.05)
General physical health deterioration*	168	2 (1.71–2.35)	1.99 (74.5)	1.88 (1.65)	0.91 (0.68)
Pancytopenia*	167	4.52 (3.8–5.37)	4.48 (355.23)	3.73 (3.23)	1.9 (1.65)
Pyrexia	164	0.9 (0.77–1.05)	0.9 (1.74)	0.91 (0.79)	−0.14 (−0.37)
Pneumonitis	132	1 (0.84–1.19)	1 (0)	1 (0.86)	0 (−0.26)
Dehydration*	131	1.25 (1.04–1.49)	1.25 (5.94)	1.23 (1.06)	0.3 (0.04)
Acute kidney injury*	131	2.28 (1.9–2.74)	2.27 (82.06)	2.12 (1.81)	1.08 (0.81)
Constipation*	122	1.5 (1.24–1.8)	1.49 (18.34)	1.45 (1.24)	0.54 (0.27)
Asthenia	119	0.88 (0.73–1.06)	0.88 (1.93)	0.88 (0.76)	−0.18 (−0.45)
Sepsis*	118	1.87 (1.54–2.26)	1.86 (42.41)	1.77 (1.51)	0.83 (0.55)
Pulmonary embolism*	115	1.32 (1.09–1.6)	1.32 (8.22)	1.29 (1.1)	0.37 (0.09)
Infection*	98	2.54 (2.05–3.15)	2.53 (78.91)	2.33 (1.95)	1.22 (0.91)
Decreased appetite	97	0.63 (0.51–0.77)	0.63 (20.45)	0.64 (0.54)	−0.63 (−0.93)
Platelet count decreased*	97	1.36 (1.11–1.68)	1.36 (8.56)	1.33 (1.12)	0.41 (0.11)
Mucosal inflammation*	93	2.72 (2.18–3.39)	2.71 (86.4)	2.47 (2.05)	1.3 (0.98)
Interstitial lung disease	91	0.62 (0.5–0.76)	0.62 (20.41)	0.64 (0.53)	−0.65 (−0.96)
Respiratory failure	88	1.02 (0.82–1.26)	1.02 (0.02)	1.01 (0.85)	0.02 (−0.3)
Rash	84	0.37 (0.3–0.46)	0.37 (87.66)	0.39 (0.32)	−1.37 (−1.69)
Abdominal pain*	83	1.49 (1.19–1.87)	1.49 (12.25)	1.45 (1.2)	0.53 (0.21)
Alopecia*	80	2.02 (1.6–2.54)	2.01 (36.26)	1.9 (1.56)	0.93 (0.59)
White blood cell count decreased*	76	1.32 (1.04–1.67)	1.32 (5.42)	1.29 (1.06)	0.37 (0.03)
Hypotension*	75	1.51 (1.19–1.91)	1.5 (11.62)	1.46 (1.2)	0.55 (0.2)
Cough	74	0.89 (0.7–1.12)	0.89 (1)	0.89 (0.73)	−0.16 (−0.5)
Hypokalaemia*	73	1.96 (1.53–2.49)	1.95 (30.32)	1.85 (1.51)	0.89 (0.53)
Hepatitis*	70	3.05 (2.36–3.93)	3.04 (80.8)	2.72 (2.19)	1.44 (1.07)
Haemoptysis	69	1.13 (0.88–1.44)	1.13 (0.92)	1.12 (0.91)	0.16 (−0.2)
Polyneuropathy*	68	8.46 (6.31–11.34)	8.43 (293.96)	5.9 (4.62)	2.56 (2.16)
Neuropathy peripheral*	65	1.78 (1.38–2.3)	1.77 (19.89)	1.7 (1.37)	0.76 (0.39)
Neutropenic sepsis*	62	4.5 (3.4–5.97)	4.49 (132)	3.74 (2.95)	1.9 (1.5)
Dysphagia*	61	1.37 (1.05–1.78)	1.36 (5.51)	1.34 (1.07)	0.42 (0.04)
Chest pain*	60	1.39 (1.07–1.81)	1.39 (6.07)	1.36 (1.09)	0.44 (0.06)
Hemoglobin decreased*	60	1.37 (1.05–1.78)	1.37 (5.52)	1.34 (1.07)	0.42 (0.04)
Pleural effusion	59	0.53 (0.41–0.69)	0.53 (23.57)	0.55 (0.44)	−0.87 (−1.25)
Pruritus	58	1 (0.77–1.31)	1 (0)	1 (0.8)	0.01 (−0.38)
Stomatitis	58	1.01 (0.78–1.32)	1.01 (0.01)	1.01 (0.81)	0.02 (−0.37)
Hypothyroidism*	57	1.36 (1.04–1.78)	1.36 (5.01)	1.33 (1.06)	0.41 (0.02)
Dizziness	55	0.91 (0.69–1.19)	0.91 (0.5)	0.91 (0.73)	−0.13 (−0.53)
Atrial fibrillation	53	1.31 (0.99–1.73)	1.31 (3.56)	1.28 (1.02)	0.36 (−0.05)
Neutrophil count decreased	53	0.98 (0.74–1.29)	0.98 (0.02)	0.98 (0.78)	−0.03 (−0.43)
Oedema peripheral	51	0.89 (0.67–1.18)	0.89 (0.66)	0.9 (0.71)	−0.16 (−0.57)

Asterisks (*) indicate statistically significant signals in algorithm; ROR, reporting odds ratio; PRR, proportional reporting ratio; EBGM, empirical Bayesian geometric mean; EBGM05, the lower limit of the 95% CI of EBGM; IC, information component; IC025, the lower limit of the 95% CI of the IC; CI, confidence interval; PT, preferred term.

### 3.4 Subgroup analysis

The subgroup analysis revealed distinct differences in carboplatin-related adverse events across sex and age groups. Among the 50 most common adverse events showing positive signals, male patients were more likely to experience gastrointestinal reactions (e.g., vomiting, dehydration), circulatory symptoms (hypotension, atrial fibrillation), and endocrine dysfunction (hypothyroidism). In contrast, female patients showed higher rates of liver dysfunction (e.g., hepatitis, hepatic failure), elevated thrombotic risk (pulmonary embolism), and immune-related reactions (hypersensitivity, immune-mediated enterocolitis). More detailed data can be found in Supplementary Tables S4, S5.

Age-stratified analysis indicated that only 13 patients were under 18 years of age, and no novel adverse events were observed in this group. In patients aged 18 to 64.9 years, there was a higher risk of hematologic events (e.g., decreased white blood cell count, decreased platelet count) and endocrine abnormalities (hypothyroidism), suggesting a more pronounced impact of chemotherapy on hematopoiesis and hormonal regulation. Patients aged 65 years or older were more prone to vomiting, constipation, dehydration, sepsis, and septic shock (see Supplementary Tables S6–S8).

### 3.5 Sensitivity analysis

Carboplatin is frequently combined with Gemcitabine, Pemetrexed, Docetaxel, Paclitaxel, Vinorelbine, Pembrolizumab, Atezolizumab, and Amivantamab in clinical practice. Excluding reports with additional concomitant medications yielded 619 reports involving 1,945 adverse events. The analysis shows that the following adverse events remain prevalent when carboplatin is used alongside these primary chemotherapeutic and immunotherapeutic agents: ANAEMIA, THROMBOCYTOPENIA, NEUTROPENIA, FEBRILE NEUTROPENIA, LEUKOPENIA, DEHYDRATION, INFECTION, PLATELET COUNT DECREASED, WHITE BLOOD CELL COUNT DECREASED, HYPOTENSION, HYPOKALAEMIA, NEUTROPENIC SEPSIS, DYSPHAGIA, CHEST PAIN, and HAEMOGLOBIN DECREASED (see Supplementary Table S9).

### 3.6 Onset of adverse events and Weibull distribution analysis

**Figure 3:**
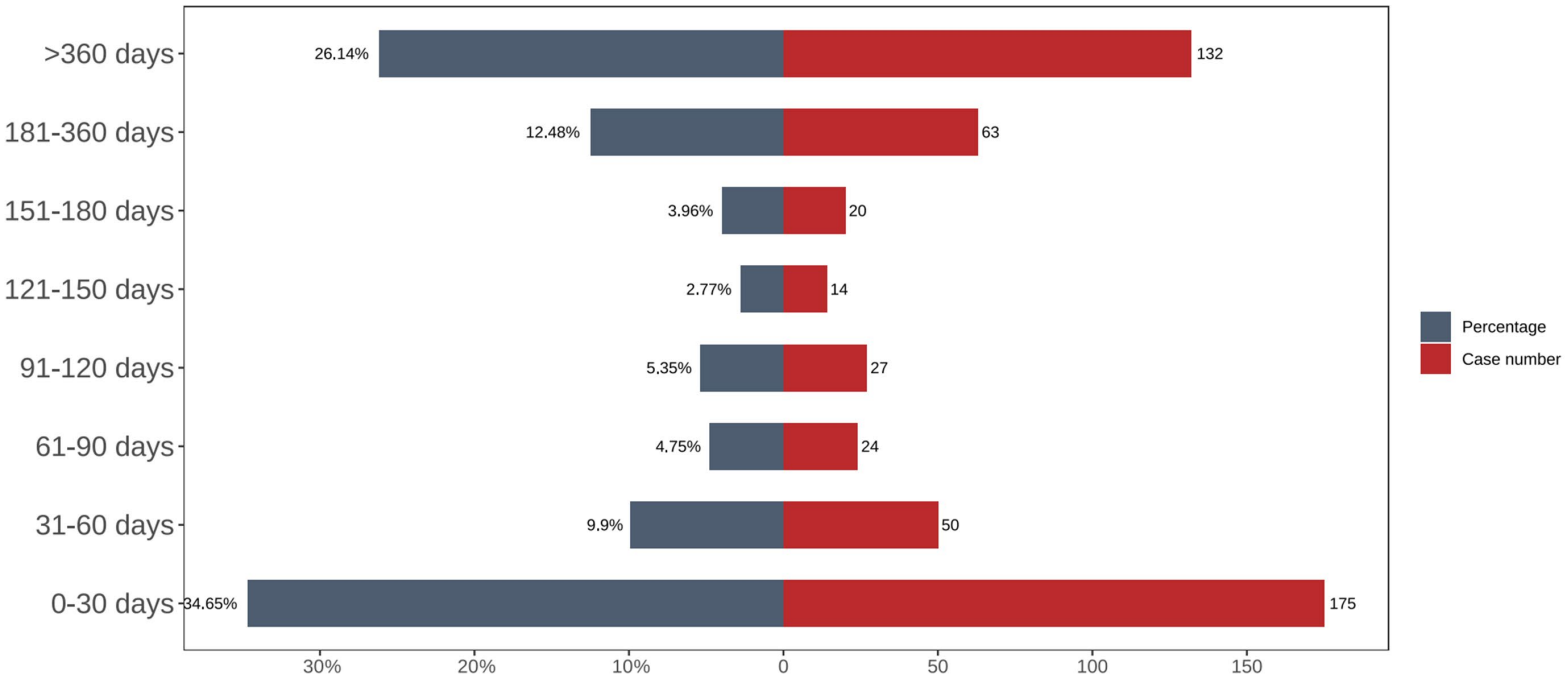
Time to onset of Carboplatin-induced adverse events.

**Figure 4:**
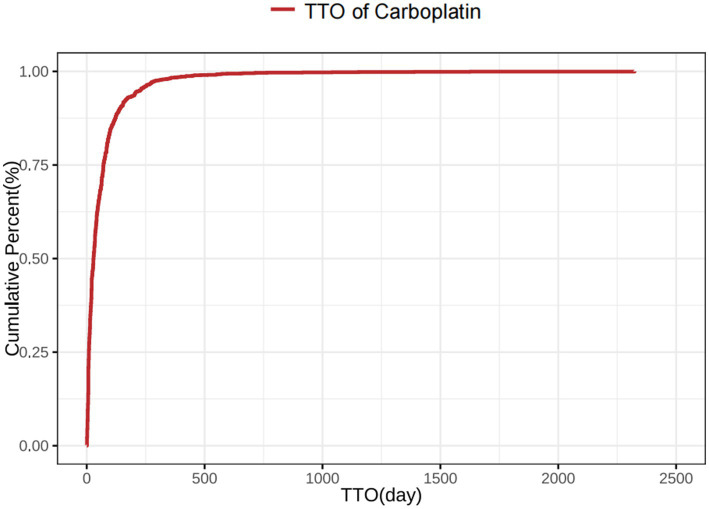
Cumulative incidence of Carboplatin-related adverse events over time.

The time-distribution analysis demonstrated that most carboplatin-related adverse events occurred within the first 30 days post-administration. Weibull distribution modeling further confirmed this early failure pattern, as illustrated in Figures 3, 4, with corresponding parameters listed in Table 4.

**Table 4:** Time to onset of Carboplatin-associated adverse events and Weibull distribution analysis.

Drug	TTO(days)		Weibull distribution		
	Case reports	Median(d)(IQR)	Scale parameter: α(95%CI)	Shape parameter: β(95%CI)	Type
Carboplatin	1848	29 (9–71)	51.72 (48.30–54.94)	0.78 (0.75–0.80)	Early failure

TTO, time to onset; CI, confidence interval; IQR, interquartile range.

## 4 Discussion

This study, conducted using the FAERS database, systematically evaluated carboplatin’s real-world safety profile in non–small cell lung cancer (NSCLC). The findings corroborated several known adverse reactions previously documented in both the literature and drug labeling—namely, anemia, neutropenia, nausea and vomiting, and renal impairment. In addition, this study identified multiple potential adverse events requiring further attention, such as abdominal pain, neutropenic sepsis, and hypothyroidism. These results underscore the need for heightened pharmacovigilance, especially within the first 30 days of treatment, to facilitate early detection and management of adverse reactions.

This study shows that ABDOMINAL PAIN not only exhibited a positive signal in the overall population but was also more pronounced among female patients. A Phase I pharmacokinetic study by Tournoux-Facon et al. (21) further suggested that carboplatin combined with other chemotherapeutic agents may induce severe ABDOMINAL PAIN, frequently accompanied by bone marrow suppression. Additional studies propose that ABDOMINAL PAIN may stem from gastrointestinal mucosal injury, concomitant infection, or abdominal aortic complications; without prompt intervention, progression to an acute abdomen is likely (22, 23).

Notably, in patients receiving chemotherapy, ABDOMINAL PAIN is frequently linked to disruption of the gastrointestinal mucosal barrier, intestinal dysbiosis, or potential infectious peritonitis; platinum-based drugs may exacerbate mucosal injury, making the gastrointestinal tract more susceptible to pathogens or triggering chemotherapy-associated enteritis (24, 25). Additionally, when chemotherapy is combined with immunotherapy, close monitoring is essential for immune-mediated colitis or even potential intestinal perforation (26, 27). Therefore, in female, older, or comorbid patients, persistent or worsening ABDOMINAL PAIN warrants immediate imaging and laboratory evaluations to rule out more severe complications and ensure timely intervention.

Neutropenic sepsis—a severe infection secondary to bone marrow suppression—also showed a high incidence and mortality rate in patients treated with carboplatin for NSCLC (21, 28, 29). In this study, incidence was notably elevated among older adults (≥65 years) and male patients. Brahmer et al. reported parallel findings when investigating carboplatin combined with bevacizumab, indicating that elderly men with decreased bone marrow reserves or multiple comorbidities may be particularly vulnerable to severe infections (30). Consequently, early preventive measures (<30 days post-chemotherapy) remain essential, including frequent blood count monitoring, prophylactic administration of granulocyte colony–stimulating factor (G-CSF), and immediate intervention at the first indication of fever (23, 31, 32).

Another significant finding of this study is the strengthened association between CARBOPLATIN and HYPOTHYROIDISM. Platinum-based agents can lower hormone secretion by directly suppressing thyroid cells or interfering with thyroid hormone synthesis pathways (33, 34). Furthermore, with the increasing use of immune checkpoint inhibitors (ICIs) in NSCLC treatment, immune activation from chemoimmunotherapy can trigger autoimmune responses or lymphocyte-mediated endocrine gland damage, resulting in subclinical or clinical HYPOTHYROIDISM (35, 36).

Previous studies indicate that immunotherapy-induced hypothyroidism is more likely with prior chemotherapy, suggesting that CARBOPLATIN may have a synergistic or potentiating effect (37, 38). In the IMpower133 trial, Mansfield et al. (37) reported endocrine-related adverse events, including thyroid insufficiency, following Atezolizumab combined with CARBOPLATIN/etoposide. Similarly, Nakagami et al. (38) documented pituitary insufficiency and resulting thyroid dysfunction in patients after immunochemotherapy.

It is therefore advisable to routinely monitor TSH, T3, and T4 from baseline through early and mid-treatment when CARBOPLATIN is combined with ICIs. Prompt intervention at the first sign of hypothyroidism is recommended to prevent severe complications such as myxedema crisis (23, 38, 39). In summary, thyroid dysfunction arising from combined chemotherapy and immunotherapy warrants heightened clinical vigilance. Early recognition and prompt management of these abnormalities are essential for improving patient outcomes and ensuring continuity of care.

Nevertheless, a critical limitation of this study lies in the challenge of attributing specific adverse events to carboplatin, particularly when it is administered in combination regimens. Agents such as taxanes (e.g., paclitaxel, docetaxel) are more commonly associated with significant alopecia, while cisplatin is more notorious for causing severe peripheral neuropathy. Accordingly, some events identified in our dataset may not originate from carboplatin itself. The most reliable method to investigate carboplatin’s intrinsic safety profile would be to analyze data from cases where carboplatin was administered as monotherapy. However, such data in large-scale databases is limited, as monotherapy for advanced NSCLC is relatively uncommon in routine clinical practice.

In clinical practice, a “one-size-fits-all” approach is commonly used for carboplatin-related adverse event monitoring. However, our data emphasize distinct risk patterns across different demographic strata. For example, female patients seem more prone to abdominal pain and hepatic dysfunction, whereas male patients appear more susceptible to infections (e.g., neutropenic sepsis) and endocrine abnormalities (e.g., hypothyroidism) (40, 41). Older adults experience an elevated risk of multisystem adverse events, contributing to higher rates of treatment discontinuation or mortality (41–43). Consequently, personalized dosing strategies are recommended, incorporating factors such as sex, age, medical history, and concurrent therapies. Moreover, closer surveillance of blood counts, hepatic and renal function, and endocrine indicators is crucial, particularly for older patients or those with poor performance status (PS).

This study used the FDA Adverse Event Reporting System (FAERS) to identify and evaluate potential safety signals of CARBOPLATIN. While this large-scale, spontaneous reporting system does capture emerging safety issues in a wide patient population, several inherent limitations must be acknowledged.

First, as a spontaneous reporting database, FAERS is prone to underreporting, selection bias, and inconsistencies in report quality. Key clinical details (e.g., dosages, concomitant medications, and baseline patient status) are often missing (21, 32). Additionally, reporting completeness and accuracy may vary based on whether healthcare professionals, patients, or pharmacists are submitting the information. Some adverse events may go unrecognized or unreported when symptoms are atypical.

Second, because FAERS lacks a control group and standardized reporting criteria, disproportionate analyses can only generate safety signals rather than prove causal relationships. Additional research—such as prospective cohort studies or large-scale real-world matching studies—is necessary to confirm the true relationship between CARBOPLATIN and the reported adverse events (22, 30). In addition, spontaneous reporting systems often highlight severe or novel events, potentially inflating the perceived clinical importance of certain rare adverse events while overlooking more common but less severe ones.

Because the FAERS dataset on concomitant treatments is incomplete, residual confounding cannot be fully excluded, even though sensitivity analyses addressed data duplication and possible confounding. Consequently, these findings remain exploratory, and further validation in larger or more heterogeneous real-world datasets is needed.

To mitigate the recognized biases of spontaneous reporting systems and enhance causal inferences concerning CARBOPLATIN in NSCLC, future research could: (1) compare outcomes with those from other major pharmacovigilance databases (e.g., EudraVigilance, WHO VigiBase) or large multicenter electronic health record systems; (2) conduct prospective cohort studies or extensive retrospective real-world analyses to gather more complete baseline characteristics, treatment regimens, and follow-up data, employing multivariable regression or propensity score matching to improve causal inference; and (3) develop refined prospective monitoring strategies or functional assessments for high-risk populations (e.g., older adults) to enable earlier, more accurate detection and intervention of adverse events.

Despite these limitations, by leveraging the extensive coverage of FAERS, this study successfully identified and quantified numerous underrecognized safety signals of CARBOPLATIN, thereby offering valuable insights for clinical management and pharmacovigilance.

This study identified multiple potentially high-risk adverse events and observed noteworthy differences across patient subgroups, leading to the following clinical monitoring and management recommendations. First, because adverse events frequently occur within 30 days of CARBOPLATIN administration—particularly during the initial 2–4 weeks—more frequent laboratory assessments (including complete blood counts, liver and kidney function tests, electrolyte levels, and thyroid function) are recommended. Persistent or worsening ABDOMINAL PAIN, fatigue, palpitations, fever, or edema should lead to immediate imaging and further evaluations.

Male patients require particular vigilance for NEUTROPENIA and HYPOTHYROIDISM, whereas female patients should be closely monitored for liver function, abdominal symptoms, and fluid–electrolyte balance. For older adults (≥65 years), a comprehensive geriatric assessment (CGA) is advised before initiating CARBOPLATIN, with dose adjustments according to renal function and comorbidities, alongside proactive measures to prevent bone marrow suppression and infection. When CARBOPLATIN is combined with immune checkpoint inhibitors (ICIs) or other cytotoxic agents, the cumulative toxicity of all treatments should be carefully assessed. Close observation of immune-related adverse events (e.g., THYROIDITIS, HEPATITIS, DERMATITIS) and thrombotic or hemorrhagic complications is crucial. In cases of ≥Grade 3 adverse events or repeated toxicity, dose reduction, extended dosing intervals, or alternative combination regimens should be considered promptly to avert cumulative toxicity.

By customizing monitoring strategies to each patient’s profile and managing the complexities of multi-agent regimens, the therapeutic benefits of CARBOPLATIN can be optimized while maintaining patient safety, ultimately delivering a more precise and secure treatment paradigm for NSCLC.

In conclusion, while carboplatin remains indispensable in treating NSCLC, vigilance over its multisystem adverse effects is crucial, particularly regarding abdominal pain, neutropenic sepsis, and hypothyroidism. Clinicians should adjust management strategies based on patient demographics and treatment contexts, and enhance monitoring and interventions when carboplatin is combined with immunotherapies or other novel agents. Such measures may improve patient outcomes and inform more precise safety guidelines for carboplatin use.

## 5 Conclusion

This study leveraged the FAERS database to examine carboplatin-related adverse event reports in NSCLC from the first quarter of 2004 through the third quarter of 2024. The findings corroborated established adverse reactions—such as bone marrow suppression, renal impairment, and gastrointestinal toxicity—and additionally revealed several potential events underemphasized on current product labeling, including abdominal pain, neutropenic sepsis, and hypothyroidism. Nonetheless, it is important to acknowledge that some adverse events like alopecia and pronounced neuropathy are more classically associated with other agents, highlighting the inherent complexity of attributing these toxicities solely to carboplatin. Subgroup analyses showed that male patients exhibited elevated risks for infection-related and endocrine events, whereas female patients were more susceptible to gastrointestinal and hepatic complications. Furthermore, patients aged ≥65 years demonstrated a greater likelihood of multisystem involvement, particularly affecting hematologic and renal function.

On the basis of these observations, the following clinical recommendations are suggested: (1) in men aged ≥65 years, baseline assessment of bone marrow reserve is crucial, and prophylactic G-CSF may be warranted to reduce neutropenic sepsis; and (2) in female patients, regular liver function monitoring (e.g., ALT, AST, bilirubin) is advised to identify drug-induced liver injury at an early stage. Future investigations focusing on carboplatin monotherapy cohorts may offer a more definitive evaluation of its individual safety profile, minimizing confounding effects from combination therapies. Such targeted measures may help refine personalized treatment regimens. Overall, the real-world safety signals elucidated by this large-scale data analysis underscore the necessity of updating guidelines for carboplatin use in NSCLC, with the objective of strengthening both efficacy and safety.

## Data Availability

The datasets presented in this study can be found in online repositories. The names of the repository/repositories and accession number(s) can be found in the article/Supplementary material.
