# Supplementary material for: Real-world safety of carboplatin in non-small cell lung cancer: a retrospective signal detection and subgroup analysis based on the FAERS database
Source: Front Med (Lausanne). 2025 Jun 16;12:1590738. doi: 10.3389/fmed.2025.1590738 (PMC12206876; doi:10.3389/fmed.2025.1590738)
Supplement: Supplementary file 1 [file Data_Sheet_1.docx]

Supplementary Material

Supplementary Tables

Supplementary Table 1:

Two-by-two contingency table for disproportionality analyses.

|  | Target AEs | Other AEs | Total |
| --- | --- | --- | --- |
| Carboplatin | a | b | a+b |
| Other drugs | c | d | c+d |
| Total | a+c | b+d | a+b+c+d |

Abbreviation: AEs, adverse events; a, number of reports containing both the target drug and target adverse drug reaction; b, number of reports containing other adverse drug reaction of the target drug; c, number of reports containing the target adverse drug reaction of other drugs; d, number of reports containing other drugs and other adverse drug reactions.

Supplementary Table 2:

Four major algorithms used for signal detection.

| Algorithms | Equation | Criteria |
| --- | --- | --- |
| ROR | ROR=ad/b/c | lower limit of 95% CI>1, N≥3 |
|  | 95%CI=e^ln(ROR)±1.96(1/a+1/b+1/c+1/d)^0.5^ |  |
| PRR | PRR=a(c+d)/c/(a+b) | PRR≥2, χ^2^≥4, N≥3 |
|  | χ^2^=[(ad-bc)^2](a+b+c+d)/[(a+b)(c+d)(a+c)(b+d)] |  |
| BCPNN | IC=log_2_a(a+b+c+d)(a+c)(a+b) | IC025>0 |
|  | 95%CI= E(IC) ± 2V(IC)^0.5 |  |
| MGPS | EBGM=a(a+b+c+d)/(a+c)/(a+b) | EBGM05>2 |
|  | 95%CI=e^ln(EBGM)±1.96(1/a+1/b+1/c+1/d)^0.5^ |  |

Abbreviation: a, number of reports containing both the target drug and target adverse drug reaction; b, number of reports containing other adverse drug reaction of the target drug; c, number of reports containing the target adverse drug reaction of other drugs; d, number of reports containing other drugs and other adverse drug reactions. 95%CI, 95% confidence interval; N, the number of reports; χ2, chi-squared; IC, information component; IC025, the lower limit of 95% CI of the IC; E(IC), the IC expectations; V(IC), the variance of IC; EBGM, empirical Bayesian geometric mean; EBGM05, the lower limit of 95% CI of EBGM.

Supplementary Table 3 :

All adverse events meeting the positive signal threshold at the PT level from FAERS data

| PT | Case numbers | ROR(95%Cl) | PRR(χ2) | EBGM(EBGM05) | IC(IC025) |
| --- | --- | --- | --- | --- | --- |
| Anaemia | 367 | 2.41 ( 2.15 - 2.69 ) | 2.37 ( 256.96 ) | 2.2 ( 2 ) | 1.14 ( 0.97 ) |
| Pneumonia | 279 | 1.35 ( 1.2 - 1.53 ) | 1.35 ( 23.23 ) | 1.32 ( 1.19 ) | 0.4 ( 0.22 ) |
| Nausea | 277 | 1.28 ( 1.13 - 1.45 ) | 1.28 ( 15.56 ) | 1.26 ( 1.13 ) | 0.33 ( 0.15 ) |
| Fatigue | 246 | 1.26 ( 1.11 - 1.44 ) | 1.26 ( 12.17 ) | 1.24 ( 1.11 ) | 0.31 ( 0.12 ) |
| Thrombocytopenia | 240 | 2.63 ( 2.3 - 3.02 ) | 2.61 ( 206.68 ) | 2.39 ( 2.13 ) | 1.26 ( 1.06 ) |
| Neutropenia | 224 | 2.19 ( 1.9 - 2.52 ) | 2.17 ( 125.77 ) | 2.03 ( 1.81 ) | 1.02 ( 0.82 ) |
| Febrile neutropenia | 210 | 2.26 ( 1.96 - 2.62 ) | 2.25 ( 128.57 ) | 2.1 ( 1.86 ) | 1.07 ( 0.86 ) |
| Leukopenia | 194 | 4.5 ( 3.83 - 5.27 ) | 4.45 ( 409.42 ) | 3.71 ( 3.25 ) | 1.89 ( 1.66 ) |
| General physical health deterioration | 168 | 2 ( 1.71 - 2.35 ) | 1.99 ( 74.5 ) | 1.88 ( 1.65 ) | 0.91 ( 0.68 ) |
| Pancytopenia | 167 | 4.52 ( 3.8 - 5.37 ) | 4.48 ( 355.23 ) | 3.73 ( 3.23 ) | 1.9 ( 1.65 ) |
| Dehydration | 131 | 1.25 ( 1.04 - 1.49 ) | 1.25 ( 5.94 ) | 1.23 ( 1.06 ) | 0.3 ( 0.04 ) |
| Acute kidney injury | 131 | 2.28 ( 1.9 - 2.74 ) | 2.27 ( 82.06 ) | 2.12 ( 1.81 ) | 1.08 ( 0.81 ) |
| Constipation | 122 | 1.5 ( 1.24 - 1.8 ) | 1.49 ( 18.34 ) | 1.45 ( 1.24 ) | 0.54 ( 0.27 ) |
| Sepsis | 118 | 1.87 ( 1.54 - 2.26 ) | 1.86 ( 42.41 ) | 1.77 ( 1.51 ) | 0.83 ( 0.55 ) |
| Pulmonary embolism | 115 | 1.32 ( 1.09 - 1.6 ) | 1.32 ( 8.22 ) | 1.29 ( 1.1 ) | 0.37 ( 0.09 ) |
| Infection | 98 | 2.54 ( 2.05 - 3.15 ) | 2.53 ( 78.91 ) | 2.33 ( 1.95 ) | 1.22 ( 0.91 ) |
| Platelet count decreased | 97 | 1.36 ( 1.11 - 1.68 ) | 1.36 ( 8.56 ) | 1.33 ( 1.12 ) | 0.41 ( 0.11 ) |
| Mucosal inflammation | 93 | 2.72 ( 2.18 - 3.39 ) | 2.71 ( 86.4 ) | 2.47 ( 2.05 ) | 1.3 ( 0.98 ) |
| Abdominal pain | 83 | 1.49 ( 1.19 - 1.87 ) | 1.49 ( 12.25 ) | 1.45 ( 1.2 ) | 0.53 ( 0.21 ) |
| Alopecia | 80 | 2.02 ( 1.6 - 2.54 ) | 2.01 ( 36.26 ) | 1.9 ( 1.56 ) | 0.93 ( 0.59 ) |
| White blood cell count decreased | 76 | 1.32 ( 1.04 - 1.67 ) | 1.32 ( 5.42 ) | 1.29 ( 1.06 ) | 0.37 ( 0.03 ) |
| Hypotension | 75 | 1.51 ( 1.19 - 1.91 ) | 1.5 ( 11.62 ) | 1.46 ( 1.2 ) | 0.55 ( 0.2 ) |
| Hypokalaemia | 73 | 1.96 ( 1.53 - 2.49 ) | 1.95 ( 30.32 ) | 1.85 ( 1.51 ) | 0.89 ( 0.53 ) |
| Hepatitis | 70 | 3.05 ( 2.36 - 3.93 ) | 3.04 ( 80.8 ) | 2.72 ( 2.19 ) | 1.44 ( 1.07 ) |
| Polyneuropathy | 68 | 8.46 ( 6.31 - 11.34 ) | 8.43 ( 293.96 ) | 5.9 ( 4.62 ) | 2.56 ( 2.16 ) |
| Neuropathy peripheral | 65 | 1.78 ( 1.38 - 2.3 ) | 1.77 ( 19.89 ) | 1.7 ( 1.37 ) | 0.76 ( 0.39 ) |
| Neutropenic sepsis | 62 | 4.5 ( 3.4 - 5.97 ) | 4.49 ( 132 ) | 3.74 ( 2.95 ) | 1.9 ( 1.5 ) |
| Dysphagia | 61 | 1.37 ( 1.05 - 1.78 ) | 1.36 ( 5.51 ) | 1.34 ( 1.07 ) | 0.42 ( 0.04 ) |
| Chest pain | 60 | 1.39 ( 1.07 - 1.81 ) | 1.39 ( 6.07 ) | 1.36 ( 1.09 ) | 0.44 ( 0.06 ) |
| Haemoglobin decreased | 60 | 1.37 ( 1.05 - 1.78 ) | 1.37 ( 5.52 ) | 1.34 ( 1.07 ) | 0.42 ( 0.04 ) |
| Hypothyroidism | 57 | 1.36 ( 1.04 - 1.78 ) | 1.36 ( 5.01 ) | 1.33 ( 1.06 ) | 0.41 ( 0.02 ) |
| Oesophagitis | 47 | 2.06 ( 1.52 - 2.79 ) | 2.06 ( 22.67 ) | 1.94 ( 1.5 ) | 0.95 ( 0.51 ) |
| Urinary tract infection | 46 | 1.38 ( 1.02 - 1.86 ) | 1.38 ( 4.36 ) | 1.35 ( 1.05 ) | 0.43 ( -0.01 ) |
| Epistaxis | 45 | 1.43 ( 1.05 - 1.94 ) | 1.43 ( 5.29 ) | 1.39 ( 1.08 ) | 0.48 ( 0.03 ) |
| Immune-mediated enterocolitis | 41 | 2.28 ( 1.65 - 3.17 ) | 2.28 ( 25.92 ) | 2.12 ( 1.62 ) | 1.09 ( 0.61 ) |
| Hypomagnesaemia | 40 | 2.89 ( 2.06 - 4.04 ) | 2.88 ( 41.84 ) | 2.6 ( 1.96 ) | 1.38 ( 0.89 ) |
| Hypersensitivity | 39 | 1.9 ( 1.36 - 2.64 ) | 1.89 ( 14.74 ) | 1.8 ( 1.36 ) | 0.85 ( 0.37 ) |
| Septic shock | 38 | 1.51 ( 1.08 - 2.1 ) | 1.5 ( 5.91 ) | 1.46 ( 1.11 ) | 0.55 ( 0.07 ) |
| Abdominal pain upper | 37 | 1.51 ( 1.08 - 2.11 ) | 1.51 ( 5.76 ) | 1.46 ( 1.1 ) | 0.55 ( 0.06 ) |
| Autoimmune hepatitis | 34 | 4.57 ( 3.12 - 6.68 ) | 4.56 ( 73.9 ) | 3.78 ( 2.75 ) | 1.92 ( 1.38 ) |
| Bone marrow failure | 33 | 2.05 ( 1.42 - 2.94 ) | 2.04 ( 15.67 ) | 1.93 ( 1.42 ) | 0.95 ( 0.42 ) |
| Hyperthyroidism | 33 | 1.77 ( 1.24 - 2.54 ) | 1.77 ( 9.98 ) | 1.69 ( 1.25 ) | 0.76 ( 0.24 ) |
| Lower respiratory tract infection | 32 | 2.72 ( 1.87 - 3.95 ) | 2.71 ( 29.69 ) | 2.47 ( 1.8 ) | 1.3 ( 0.77 ) |
| C-reactive protein increased | 30 | 1.57 ( 1.08 - 2.29 ) | 1.57 ( 5.72 ) | 1.52 ( 1.11 ) | 0.61 ( 0.06 ) |
| Drug eruption | 28 | 2.17 ( 1.46 - 3.22 ) | 2.17 ( 15.62 ) | 2.03 ( 1.46 ) | 1.02 ( 0.46 ) |
| Tubulointerstitial nephritis | 27 | 1.91 ( 1.28 - 2.85 ) | 1.91 ( 10.52 ) | 1.82 ( 1.3 ) | 0.86 ( 0.29 ) |
| Hypertransaminasaemia | 27 | 5.33 ( 3.45 - 8.23 ) | 5.32 ( 71.51 ) | 4.26 ( 2.96 ) | 2.09 ( 1.48 ) |
| Immune-mediated hepatitis | 27 | 3.3 ( 2.18 - 4.99 ) | 3.3 ( 35.96 ) | 2.91 ( 2.06 ) | 1.54 ( 0.95 ) |
| Cognitive disorder | 26 | 1.97 ( 1.31 - 2.96 ) | 1.97 ( 11.07 ) | 1.86 ( 1.33 ) | 0.9 ( 0.31 ) |
| Disease recurrence | 26 | 6.55 ( 4.16 - 10.33 ) | 6.54 ( 87.23 ) | 4.96 ( 3.39 ) | 2.31 ( 1.68 ) |
| Haematotoxicity | 26 | 2.5 ( 1.66 - 3.79 ) | 2.5 ( 20.34 ) | 2.3 ( 1.63 ) | 1.2 ( 0.61 ) |
| Bone pain | 26 | 1.76 ( 1.17 - 2.64 ) | 1.76 ( 7.67 ) | 1.68 ( 1.2 ) | 0.75 ( 0.17 ) |
| Intentional product use issue | 26 | 1.93 ( 1.28 - 2.89 ) | 1.92 ( 10.34 ) | 1.83 ( 1.3 ) | 0.87 ( 0.28 ) |
| Hepatic failure | 26 | 1.94 ( 1.29 - 2.92 ) | 1.94 ( 10.63 ) | 1.84 ( 1.31 ) | 0.88 ( 0.3 ) |
| Nephritis | 25 | 2.97 ( 1.94 - 4.54 ) | 2.96 ( 27.55 ) | 2.66 ( 1.86 ) | 1.41 ( 0.8 ) |
| Immune-mediated lung disease | 25 | 1.67 ( 1.11 - 2.52 ) | 1.67 ( 6.09 ) | 1.61 ( 1.14 ) | 0.68 ( 0.09 ) |
| Therapy partial responder | 24 | 1.58 ( 1.04 - 2.41 ) | 1.58 ( 4.7 ) | 1.53 ( 1.08 ) | 0.61 ( 0.01 ) |
| Lymphopenia | 24 | 3.82 ( 2.45 - 5.95 ) | 3.81 ( 40.38 ) | 3.28 ( 2.26 ) | 1.71 ( 1.08 ) |
| Immune-mediated hypothyroidism | 24 | 5.31 ( 3.35 - 8.42 ) | 5.3 ( 63.33 ) | 4.25 ( 2.89 ) | 2.09 ( 1.44 ) |
| Stevens-johnson syndrome | 23 | 2.31 ( 1.49 - 3.58 ) | 2.31 ( 14.95 ) | 2.15 ( 1.49 ) | 1.1 ( 0.48 ) |
| Prescribed overdose | 23 | 5.71 ( 3.55 - 9.18 ) | 5.7 ( 66.12 ) | 4.49 ( 3.01 ) | 2.17 ( 1.5 ) |
| Renal tubular necrosis | 22 | 7.06 ( 4.28 - 11.65 ) | 7.06 ( 79.91 ) | 5.23 ( 3.44 ) | 2.39 ( 1.7 ) |
| Myelodysplastic syndrome | 21 | 5.83 ( 3.54 - 9.59 ) | 5.82 ( 61.87 ) | 4.56 ( 3 ) | 2.19 ( 1.49 ) |
| Skin toxicity | 21 | 1.85 ( 1.18 - 2.9 ) | 1.85 ( 7.33 ) | 1.76 ( 1.21 ) | 0.82 ( 0.17 ) |
| Intestinal perforation | 20 | 2.32 ( 1.45 - 3.71 ) | 2.32 ( 13.16 ) | 2.16 ( 1.46 ) | 1.11 ( 0.44 ) |
| Therapeutic product effect incomplete | 20 | 5.28 ( 3.19 - 8.75 ) | 5.28 ( 52.43 ) | 4.23 ( 2.78 ) | 2.08 ( 1.38 ) |
| Respiratory tract infection | 19 | 2.07 ( 1.29 - 3.34 ) | 2.07 ( 9.36 ) | 1.95 ( 1.31 ) | 0.96 ( 0.28 ) |
| Gastritis | 19 | 1.71 ( 1.06 - 2.74 ) | 1.71 ( 5.05 ) | 1.64 ( 1.1 ) | 0.71 ( 0.04 ) |
| Rash maculo-papular | 19 | 1.61 ( 1.01 - 2.58 ) | 1.61 ( 4 ) | 1.56 ( 1.05 ) | 0.64 ( -0.04 ) |
| Hypercalcaemia | 19 | 1.83 ( 1.14 - 2.94 ) | 1.83 ( 6.42 ) | 1.74 ( 1.17 ) | 0.8 ( 0.12 ) |
| Hypophysitis | 19 | 2.16 ( 1.34 - 3.49 ) | 2.16 ( 10.44 ) | 2.02 ( 1.36 ) | 1.02 ( 0.33 ) |
| Agranulocytosis | 18 | 4.04 ( 2.41 - 6.76 ) | 4.03 ( 32.95 ) | 3.43 ( 2.23 ) | 1.78 ( 1.05 ) |
| Anaphylactic shock | 17 | 2.68 ( 1.6 - 4.47 ) | 2.67 ( 15.32 ) | 2.44 ( 1.59 ) | 1.29 ( 0.56 ) |
| Peripheral sensory neuropathy | 17 | 1.95 ( 1.18 - 3.22 ) | 1.94 ( 6.97 ) | 1.84 ( 1.21 ) | 0.88 ( 0.17 ) |
| Vertigo | 17 | 1.83 ( 1.11 - 3.02 ) | 1.83 ( 5.75 ) | 1.75 ( 1.15 ) | 0.8 ( 0.09 ) |
| Autoimmune haemolytic anaemia | 17 | 5.06 ( 2.94 - 8.72 ) | 5.06 ( 42.26 ) | 4.1 ( 2.6 ) | 2.03 ( 1.28 ) |
| Febrile bone marrow aplasia | 16 | 4.44 ( 2.55 - 7.72 ) | 4.44 ( 33.5 ) | 3.7 ( 2.33 ) | 1.89 ( 1.11 ) |
| Ventricular tachycardia | 16 | 3.74 ( 2.17 - 6.44 ) | 3.74 ( 26.13 ) | 3.23 ( 2.05 ) | 1.69 ( 0.93 ) |
| Candida infection | 15 | 3.66 ( 2.09 - 6.42 ) | 3.66 ( 23.72 ) | 3.17 ( 1.99 ) | 1.67 ( 0.88 ) |
| Autoimmune colitis | 15 | 4.46 ( 2.52 - 7.9 ) | 4.46 ( 31.65 ) | 3.72 ( 2.31 ) | 1.9 ( 1.1 ) |
| Ear pain | 15 | 5.22 ( 2.92 - 9.34 ) | 5.22 ( 38.8 ) | 4.2 ( 2.58 ) | 2.07 ( 1.26 ) |
| Prescribed underdose | 15 | 1.82 ( 1.07 - 3.1 ) | 1.82 ( 4.97 ) | 1.74 ( 1.11 ) | 0.8 ( 0.04 ) |
| Hepatocellular injury | 14 | 3.88 ( 2.17 - 6.96 ) | 3.88 ( 24.21 ) | 3.33 ( 2.04 ) | 1.73 ( 0.92 ) |
| Hiccups | 13 | 2.5 ( 1.4 - 4.49 ) | 2.5 ( 10.17 ) | 2.3 ( 1.41 ) | 1.2 ( 0.38 ) |
| Systemic inflammatory response syndrome | 13 | 7.09 ( 3.7 - 13.6 ) | 7.09 ( 47.43 ) | 5.25 ( 3.04 ) | 2.39 ( 1.51 ) |
| Restlessness | 13 | 3.18 ( 1.75 - 5.75 ) | 3.17 ( 16.21 ) | 2.82 ( 1.72 ) | 1.5 ( 0.66 ) |
| Hypovolaemia | 13 | 3.27 ( 1.8 - 5.94 ) | 3.27 ( 17.09 ) | 2.89 ( 1.76 ) | 1.53 ( 0.7 ) |
| Pulmonary sepsis | 12 | 5.04 ( 2.64 - 9.62 ) | 5.03 ( 29.66 ) | 4.08 ( 2.38 ) | 2.03 ( 1.13 ) |
| Oral candidiasis | 12 | 1.87 ( 1.03 - 3.4 ) | 1.87 ( 4.36 ) | 1.78 ( 1.08 ) | 0.83 ( -0.01 ) |
| Immune-mediated pancreatitis | 12 | 6.55 ( 3.35 - 12.79 ) | 6.54 ( 40.25 ) | 4.96 ( 2.83 ) | 2.31 ( 1.4 ) |
| Autoimmune nephritis | 12 | 10.91 ( 5.26 - 22.66 ) | 10.9 ( 64.78 ) | 6.94 ( 3.77 ) | 2.8 ( 1.84 ) |
| Haemophagocytic lymphohistiocytosis | 12 | 2.23 ( 1.22 - 4.08 ) | 2.23 ( 7.17 ) | 2.08 ( 1.26 ) | 1.06 ( 0.21 ) |
| Non-cardiac chest pain | 11 | 3.75 ( 1.95 - 7.22 ) | 3.75 ( 18.04 ) | 3.24 ( 1.87 ) | 1.69 ( 0.78 ) |
| Circulatory collapse | 11 | 2 ( 1.07 - 3.74 ) | 2 ( 4.9 ) | 1.89 ( 1.12 ) | 0.92 ( 0.04 ) |
| Decubitus ulcer | 11 | 3.6 ( 1.87 - 6.92 ) | 3.6 ( 16.92 ) | 3.13 ( 1.81 ) | 1.65 ( 0.74 ) |
| Febrile infection | 11 | 6.21 ( 3.1 - 12.43 ) | 6.2 ( 34.82 ) | 4.77 ( 2.67 ) | 2.25 ( 1.31 ) |
| Blood magnesium decreased | 11 | 2.34 ( 1.24 - 4.4 ) | 2.34 ( 7.36 ) | 2.17 ( 1.28 ) | 1.12 ( 0.23 ) |
| Immune-mediated hyperthyroidism | 11 | 7.83 ( 3.82 - 16.06 ) | 7.82 ( 44.28 ) | 5.62 ( 3.08 ) | 2.49 ( 1.52 ) |
| Therapeutic response decreased | 10 | 2.41 ( 1.24 - 4.67 ) | 2.41 ( 7.16 ) | 2.23 ( 1.28 ) | 1.15 ( 0.23 ) |
| Arteriosclerosis | 10 | 5.28 ( 2.59 - 10.77 ) | 5.28 ( 26.21 ) | 4.23 ( 2.33 ) | 2.08 ( 1.1 ) |
| Pulmonary alveolar haemorrhage | 9 | 2.05 ( 1.02 - 4.09 ) | 2.04 ( 4.27 ) | 1.93 ( 1.08 ) | 0.95 ( -0.02 ) |
| Lung abscess | 9 | 2.07 ( 1.04 - 4.15 ) | 2.07 ( 4.44 ) | 1.95 ( 1.09 ) | 0.97 ( 0 ) |
| Systemic lupus erythematosus | 9 | 5.26 ( 2.48 - 11.15 ) | 5.26 ( 23.48 ) | 4.22 ( 2.25 ) | 2.08 ( 1.05 ) |
| Immune-mediated thyroiditis | 9 | 4.21 ( 2.02 - 8.76 ) | 4.21 ( 17.5 ) | 3.55 ( 1.92 ) | 1.83 ( 0.82 ) |
| Superior vena cava syndrome | 8 | 2.67 ( 1.26 - 5.64 ) | 2.67 ( 7.19 ) | 2.44 ( 1.3 ) | 1.28 ( 0.25 ) |
| Squamous cell carcinoma of lung | 8 | 14.55 ( 5.61 - 37.71 ) | 14.54 ( 53.4 ) | 8.17 ( 3.68 ) | 3.03 ( 1.85 ) |
| Oesophageal stenosis | 8 | 2.18 ( 1.04 - 4.56 ) | 2.18 ( 4.51 ) | 2.04 ( 1.1 ) | 1.03 ( 0.01 ) |
| Exfoliative rash | 8 | 7.27 ( 3.16 - 16.73 ) | 7.27 ( 29.95 ) | 5.34 ( 2.66 ) | 2.42 ( 1.31 ) |
| Escherichia sepsis | 8 | 7.27 ( 3.16 - 16.73 ) | 7.27 ( 29.95 ) | 5.34 ( 2.66 ) | 2.42 ( 1.31 ) |
| Granulocytopenia | 8 | 3.44 ( 1.61 - 7.39 ) | 3.44 ( 11.46 ) | 3.02 ( 1.59 ) | 1.59 ( 0.54 ) |
| Cytomegalovirus infection | 8 | 2.3 ( 1.1 - 4.81 ) | 2.3 ( 5.13 ) | 2.14 ( 1.15 ) | 1.1 ( 0.07 ) |
| Hyperuricaemia | 8 | 2.62 ( 1.24 - 5.52 ) | 2.62 ( 6.89 ) | 2.39 ( 1.28 ) | 1.26 ( 0.23 ) |
| Pancreatic failure | 8 | 13.09 ( 5.17 - 33.18 ) | 13.09 ( 49.61 ) | 7.71 ( 3.54 ) | 2.95 ( 1.78 ) |
| Autoimmune hypothyroidism | 8 | 11.9 ( 4.79 - 29.59 ) | 11.9 ( 46.23 ) | 7.31 ( 3.41 ) | 2.87 ( 1.71 ) |
| Coronary artery thrombosis | 8 | 14.55 ( 5.61 - 37.71 ) | 14.54 ( 53.4 ) | 8.17 ( 3.68 ) | 3.03 ( 1.85 ) |
| Oesophagitis ulcerative | 8 | 9.35 ( 3.92 - 22.29 ) | 9.35 ( 37.95 ) | 6.31 ( 3.05 ) | 2.66 ( 1.52 ) |
| Large intestine infection | 8 | 9.35 ( 3.92 - 22.29 ) | 9.35 ( 37.95 ) | 6.31 ( 3.05 ) | 2.66 ( 1.52 ) |
| Blood uric acid increased | 7 | 3.18 ( 1.42 - 7.15 ) | 3.18 ( 8.76 ) | 2.83 ( 1.43 ) | 1.5 ( 0.39 ) |
| Tracheo-oesophageal fistula | 7 | 4.09 ( 1.79 - 9.37 ) | 4.09 ( 13.07 ) | 3.47 ( 1.74 ) | 1.8 ( 0.67 ) |
| Oesophageal candidiasis | 7 | 2.29 ( 1.04 - 5.05 ) | 2.29 ( 4.46 ) | 2.13 ( 1.1 ) | 1.09 ( 0 ) |
| Jugular vein thrombosis | 7 | 4.98 ( 2.14 - 11.61 ) | 4.98 ( 17.06 ) | 4.05 ( 1.99 ) | 2.02 ( 0.87 ) |
| Pallor | 7 | 2.94 ( 1.31 - 6.57 ) | 2.94 ( 7.58 ) | 2.64 ( 1.35 ) | 1.4 ( 0.3 ) |
| Cardiovascular disorder | 7 | 2.79 ( 1.25 - 6.23 ) | 2.79 ( 6.88 ) | 2.53 ( 1.29 ) | 1.34 ( 0.24 ) |
| Actinic keratosis | 7 | 22.91 ( 7.27 - 72.19 ) | 22.9 ( 61.09 ) | 10.12 ( 3.88 ) | 3.34 ( 2.04 ) |
| Faecaloma | 7 | 6.03 ( 2.53 - 14.34 ) | 6.03 ( 21.45 ) | 4.67 ( 2.26 ) | 2.22 ( 1.06 ) |
| Gastroenteritis norovirus | 7 | 57.27 ( 11.9 - 275.74 ) | 57.25 ( 85.97 ) | 13.5 ( 3.62 ) | 3.75 ( 2.39 ) |
| Diarrhoea haemorrhagic | 7 | 3.18 ( 1.42 - 7.15 ) | 3.18 ( 8.76 ) | 2.83 ( 1.43 ) | 1.5 ( 0.39 ) |
| Endocarditis | 7 | 3.69 ( 1.63 - 8.39 ) | 3.69 ( 11.22 ) | 3.2 ( 1.61 ) | 1.68 ( 0.56 ) |
| Hepatitis toxic | 7 | 4.09 ( 1.79 - 9.37 ) | 4.09 ( 13.07 ) | 3.47 ( 1.74 ) | 1.8 ( 0.67 ) |
| Disturbance in attention | 7 | 2.34 ( 1.06 - 5.16 ) | 2.34 ( 4.68 ) | 2.17 ( 1.12 ) | 1.12 ( 0.03 ) |
| Skin candida | 7 | 3.37 ( 1.49 - 7.6 ) | 3.37 ( 9.66 ) | 2.96 ( 1.5 ) | 1.57 ( 0.45 ) |
| Proctalgia | 7 | 6.74 ( 2.79 - 16.25 ) | 6.74 ( 24.22 ) | 5.06 ( 2.42 ) | 2.34 ( 1.17 ) |
| Subileus | 7 | 4.98 ( 2.14 - 11.61 ) | 4.98 ( 17.06 ) | 4.05 ( 1.99 ) | 2.02 ( 0.87 ) |
| Inflammatory marker increased | 7 | 3.95 ( 1.73 - 9.02 ) | 3.95 ( 12.42 ) | 3.37 ( 1.69 ) | 1.75 ( 0.63 ) |
| Immune-mediated nephritis | 7 | 3.47 ( 1.54 - 7.85 ) | 3.47 ( 10.15 ) | 3.04 ( 1.53 ) | 1.6 ( 0.49 ) |
| Brain natriuretic peptide increased | 6 | 4.46 ( 1.81 - 11.01 ) | 4.46 ( 12.66 ) | 3.72 ( 1.75 ) | 1.9 ( 0.68 ) |
| Portal venous gas | 6 | 14.03 ( 4.71 - 41.74 ) | 14.02 ( 39.07 ) | 8.01 ( 3.22 ) | 3 ( 1.67 ) |
| Angioedema | 6 | 2.65 ( 1.12 - 6.29 ) | 2.65 ( 5.32 ) | 2.42 ( 1.18 ) | 1.28 ( 0.1 ) |
| Neutropenic colitis | 6 | 6.14 ( 2.4 - 15.68 ) | 6.13 ( 18.75 ) | 4.73 ( 2.16 ) | 2.24 ( 1 ) |
| Contraindicated product administered | 6 | 19.64 ( 5.99 - 64.35 ) | 19.63 ( 48.22 ) | 9.47 ( 3.51 ) | 3.24 ( 1.87 ) |
| Paraneoplastic rash | 6 | 32.73 ( 8.18 - 130.87 ) | 32.71 ( 61.49 ) | 11.57 ( 3.63 ) | 3.53 ( 2.11 ) |
| Klebsiella infection | 6 | 5.17 ( 2.06 - 12.94 ) | 5.17 ( 15.32 ) | 4.17 ( 1.93 ) | 2.06 ( 0.83 ) |
| Anaemia of malignant disease | 6 | 12.27 ( 4.26 - 35.37 ) | 12.27 ( 35.48 ) | 7.44 ( 3.07 ) | 2.9 ( 1.57 ) |
| Sarcoidosis | 6 | 8.18 ( 3.07 - 21.8 ) | 8.18 ( 25.2 ) | 5.79 ( 2.55 ) | 2.53 ( 1.26 ) |
| Eyelid oedema | 6 | 3.27 ( 1.36 - 7.86 ) | 3.27 ( 7.89 ) | 2.89 ( 1.39 ) | 1.53 ( 0.34 ) |
| Extravasation | 6 | 4.27 ( 1.74 - 10.48 ) | 4.27 ( 11.9 ) | 3.59 ( 1.69 ) | 1.84 ( 0.63 ) |
| Hepatitis cholestatic | 6 | 8.92 ( 3.3 - 24.14 ) | 8.92 ( 27.31 ) | 6.13 ( 2.66 ) | 2.61 ( 1.33 ) |
| Immune system disorder | 6 | 2.52 ( 1.07 - 5.95 ) | 2.52 ( 4.75 ) | 2.31 ( 1.13 ) | 1.21 ( 0.04 ) |
| Lichen planus | 6 | 3.39 ( 1.41 - 8.15 ) | 3.38 ( 8.35 ) | 2.98 ( 1.43 ) | 1.57 ( 0.38 ) |
| Escherichia urinary tract infection | 6 | 4.67 ( 1.89 - 11.58 ) | 4.67 ( 13.48 ) | 3.86 ( 1.81 ) | 1.95 ( 0.73 ) |
| Immune-mediated hypophysitis | 6 | 4.46 ( 1.81 - 11.01 ) | 4.46 ( 12.66 ) | 3.72 ( 1.75 ) | 1.9 ( 0.68 ) |
| Hepatitis b reactivation | 6 | 4.09 ( 1.67 - 10.01 ) | 4.09 ( 11.2 ) | 3.47 ( 1.64 ) | 1.8 ( 0.59 ) |
| Bronchial fistula | 5 | 2.73 ( 1.06 - 7.03 ) | 2.73 ( 4.68 ) | 2.48 ( 1.12 ) | 1.31 ( 0.04 ) |
| Hypotonia | 5 | 4.54 ( 1.69 - 12.24 ) | 4.54 ( 10.81 ) | 3.77 ( 1.65 ) | 1.92 ( 0.6 ) |
| Aplastic anaemia | 5 | 2.64 ( 1.03 - 6.79 ) | 2.64 ( 4.38 ) | 2.41 ( 1.09 ) | 1.27 ( 0 ) |
| Metabolic encephalopathy | 5 | 5.45 ( 1.98 - 15.01 ) | 5.45 ( 13.63 ) | 4.34 ( 1.86 ) | 2.12 ( 0.78 ) |
| Gingival disorder | 5 | 6.82 ( 2.4 - 19.35 ) | 6.82 ( 17.51 ) | 5.1 ( 2.13 ) | 2.35 ( 0.99 ) |
| Salivary hypersecretion | 5 | 4.09 ( 1.53 - 10.9 ) | 4.09 ( 9.34 ) | 3.47 ( 1.53 ) | 1.8 ( 0.49 ) |
| Melanocytic naevus | 5 | 5.45 ( 1.98 - 15.01 ) | 5.45 ( 13.63 ) | 4.34 ( 1.86 ) | 2.12 ( 0.78 ) |
| Skin plaque | 5 | 11.69 ( 3.71 - 36.83 ) | 11.68 ( 28.49 ) | 7.23 ( 2.77 ) | 2.85 ( 1.43 ) |
| Cardiac failure chronic | 5 | 3.03 ( 1.17 - 7.87 ) | 3.03 ( 5.73 ) | 2.71 ( 1.22 ) | 1.44 ( 0.16 ) |
| Subclavian artery occlusion | 5 | 40.9 ( 7.94 - 210.86 ) | 40.89 ( 55.6 ) | 12.4 ( 3.14 ) | 3.63 ( 2.07 ) |
| Oral fungal infection | 5 | 3.15 ( 1.21 - 8.19 ) | 3.15 ( 6.14 ) | 2.8 ( 1.26 ) | 1.49 ( 0.2 ) |
| Duodenitis | 5 | 2.73 ( 1.06 - 7.03 ) | 2.73 ( 4.68 ) | 2.48 ( 1.12 ) | 1.31 ( 0.04 ) |
| Alpha haemolytic streptococcal infection | 5 | 20.45 ( 5.49 - 76.17 ) | 20.45 ( 41.1 ) | 9.64 ( 3.21 ) | 3.27 ( 1.78 ) |
| Metastases to skin | 5 | 4.09 ( 1.53 - 10.9 ) | 4.09 ( 9.34 ) | 3.47 ( 1.53 ) | 1.8 ( 0.49 ) |
| Staphylococcal sepsis | 5 | 2.73 ( 1.06 - 7.03 ) | 2.73 ( 4.68 ) | 2.48 ( 1.12 ) | 1.31 ( 0.04 ) |
| Urinary tract infection enterococcal | 5 | 5.45 ( 1.98 - 15.01 ) | 5.45 ( 13.63 ) | 4.34 ( 1.86 ) | 2.12 ( 0.78 ) |
| Glomerulonephritis rapidly progressive | 5 | 7.44 ( 2.58 - 21.41 ) | 7.43 ( 19.15 ) | 5.42 ( 2.24 ) | 2.44 ( 1.07 ) |
| Troponin t increased | 5 | 3.41 ( 1.3 - 8.93 ) | 3.41 ( 7.04 ) | 2.99 ( 1.34 ) | 1.58 ( 0.29 ) |
| Gingival pain | 5 | 2.92 ( 1.13 - 7.57 ) | 2.92 ( 5.36 ) | 2.63 ( 1.19 ) | 1.39 ( 0.12 ) |
| Chronic myelomonocytic leukaemia | 5 | 40.9 ( 7.94 - 210.86 ) | 40.89 ( 55.6 ) | 12.4 ( 3.14 ) | 3.63 ( 2.07 ) |
| Cardiovascular insufficiency | 4 | 3.85 ( 1.3 - 11.44 ) | 3.85 ( 6.83 ) | 3.31 ( 1.33 ) | 1.73 ( 0.3 ) |
| Peroneal nerve palsy | 4 | 4.09 ( 1.37 - 12.24 ) | 4.09 ( 7.47 ) | 3.47 ( 1.39 ) | 1.8 ( 0.36 ) |
| Cold sweat | 4 | 4.09 ( 1.37 - 12.24 ) | 4.09 ( 7.47 ) | 3.47 ( 1.39 ) | 1.8 ( 0.36 ) |
| Emphysematous pyelonephritis | 4 | 21.81 ( 4.88 - 97.48 ) | 21.81 ( 34.04 ) | 9.92 ( 2.83 ) | 3.31 ( 1.66 ) |
| Pneumonia klebsiella | 4 | 5.45 ( 1.76 - 16.91 ) | 5.45 ( 10.91 ) | 4.34 ( 1.68 ) | 2.12 ( 0.65 ) |
| Abdominal mass | 4 | 10.91 ( 3.08 - 38.66 ) | 10.9 ( 21.59 ) | 6.94 ( 2.41 ) | 2.8 ( 1.24 ) |
| Small intestine adenocarcinoma | 4 | Inf ( NaN - Inf ) | Inf ( 65.43 ) | 17.36 ( 0 ) | 4.12 ( 2.29 ) |
| Klebsiella sepsis | 4 | 8.18 ( 2.46 - 27.17 ) | 8.18 ( 16.8 ) | 5.79 ( 2.12 ) | 2.53 ( 1.01 ) |
| Staphylococcus test positive | 4 | 4.36 ( 1.45 - 13.15 ) | 4.36 ( 8.18 ) | 3.65 ( 1.45 ) | 1.87 ( 0.43 ) |
| Demyelinating polyneuropathy | 4 | 6.54 ( 2.05 - 20.87 ) | 6.54 ( 13.42 ) | 4.96 ( 1.88 ) | 2.31 ( 0.82 ) |
| Peripheral embolism | 4 | 3.64 ( 1.23 - 10.74 ) | 3.63 ( 6.25 ) | 3.16 ( 1.27 ) | 1.66 ( 0.23 ) |
| Necrotising myositis | 4 | 10.91 ( 3.08 - 38.66 ) | 10.9 ( 21.59 ) | 6.94 ( 2.41 ) | 2.8 ( 1.24 ) |
| Serratia infection | 4 | 16.36 ( 4.09 - 65.43 ) | 16.36 ( 28.84 ) | 8.68 ( 2.72 ) | 3.12 ( 1.51 ) |
| Leg amputation | 4 | 32.72 ( 5.99 - 178.67 ) | 32.71 ( 40.99 ) | 11.57 ( 2.8 ) | 3.53 ( 1.84 ) |
| Lymph node pain | 4 | 16.36 ( 4.09 - 65.43 ) | 16.36 ( 28.84 ) | 8.68 ( 2.72 ) | 3.12 ( 1.51 ) |
| Myelitis transverse | 4 | 5.95 ( 1.89 - 18.69 ) | 5.95 ( 12.07 ) | 4.63 ( 1.78 ) | 2.21 ( 0.73 ) |
| Aphthous ulcer | 4 | 2.85 ( 0.98 - 8.23 ) | 2.84 ( 4.08 ) | 2.57 ( 1.06 ) | 1.36 ( -0.04 ) |
| Oligoastrocytoma | 4 | 21.81 ( 4.88 - 97.48 ) | 21.81 ( 34.04 ) | 9.92 ( 2.83 ) | 3.31 ( 1.66 ) |
| Anaemia of chronic disease | 4 | 7.27 ( 2.24 - 23.61 ) | 7.27 ( 14.97 ) | 5.34 ( 1.99 ) | 2.42 ( 0.91 ) |
| Streptococcal infection | 4 | 3.85 ( 1.3 - 11.44 ) | 3.85 ( 6.83 ) | 3.31 ( 1.33 ) | 1.73 ( 0.3 ) |
| Thermal burn | 4 | 4.36 ( 1.45 - 13.15 ) | 4.36 ( 8.18 ) | 3.65 ( 1.45 ) | 1.87 ( 0.43 ) |
| Wound necrosis | 4 | 32.72 ( 5.99 - 178.67 ) | 32.71 ( 40.99 ) | 11.57 ( 2.8 ) | 3.53 ( 1.84 ) |
| Vascular pseudoaneurysm | 3 | 9.82 ( 2.35 - 41.08 ) | 9.81 ( 14.84 ) | 6.51 ( 1.96 ) | 2.7 ( 0.97 ) |
| Peritonitis bacterial | 3 | 12.27 ( 2.75 - 54.83 ) | 12.27 ( 17.74 ) | 7.44 ( 2.13 ) | 2.9 ( 1.13 ) |
| Tracheal fistula | 3 | 4.09 ( 1.15 - 14.49 ) | 4.09 ( 5.6 ) | 3.47 ( 1.2 ) | 1.8 ( 0.18 ) |
| Pseudomonas test positive | 3 | 9.82 ( 2.35 - 41.08 ) | 9.81 ( 14.84 ) | 6.51 ( 1.96 ) | 2.7 ( 0.97 ) |
| Systemic candida | 3 | 4.09 ( 1.15 - 14.49 ) | 4.09 ( 5.6 ) | 3.47 ( 1.2 ) | 1.8 ( 0.18 ) |
| Emphysematous cystitis | 3 | 6.13 ( 1.63 - 23.13 ) | 6.13 ( 9.38 ) | 4.73 ( 1.56 ) | 2.24 ( 0.58 ) |
| Kidney fibrosis | 3 | 7.01 ( 1.81 - 27.12 ) | 7.01 ( 10.82 ) | 5.21 ( 1.68 ) | 2.38 ( 0.7 ) |
| Pulmonary sarcoidosis | 3 | 8.18 ( 2.05 - 32.71 ) | 8.18 ( 12.6 ) | 5.79 ( 1.81 ) | 2.53 ( 0.83 ) |
| Clostridial infection | 3 | 4.09 ( 1.15 - 14.49 ) | 4.09 ( 5.6 ) | 3.47 ( 1.2 ) | 1.8 ( 0.18 ) |
| Chronic fatigue syndrome | 3 | 16.36 ( 3.3 - 81.06 ) | 16.36 ( 21.63 ) | 8.68 ( 2.27 ) | 3.12 ( 1.31 ) |
| Dysaesthesia | 3 | 4.91 ( 1.35 - 17.83 ) | 4.91 ( 7.18 ) | 4.01 ( 1.36 ) | 2 ( 0.37 ) |
| Streptococcal sepsis | 3 | 9.82 ( 2.35 - 41.08 ) | 9.81 ( 14.84 ) | 6.51 ( 1.96 ) | 2.7 ( 0.97 ) |
| Vascular access site swelling | 3 | Inf ( NaN - Inf ) | Inf ( 49.07 ) | 17.36 ( 0 ) | 4.12 ( 2.08 ) |
| Ear swelling | 3 | 16.36 ( 3.3 - 81.06 ) | 16.36 ( 21.63 ) | 8.68 ( 2.27 ) | 3.12 ( 1.31 ) |
| Varices oesophageal | 3 | 4.46 ( 1.24 - 15.99 ) | 4.46 ( 6.33 ) | 3.72 ( 1.28 ) | 1.9 ( 0.27 ) |
| Parvovirus b19 test positive | 3 | 49.08 ( 5.1 - 471.87 ) | 49.07 ( 35.32 ) | 13.02 ( 1.96 ) | 3.7 ( 1.77 ) |
| Roseolovirus test positive | 3 | 49.08 ( 5.1 - 471.87 ) | 49.07 ( 35.32 ) | 13.02 ( 1.96 ) | 3.7 ( 1.77 ) |
| Blood creatine phosphokinase abnormal | 3 | 6.13 ( 1.63 - 23.13 ) | 6.13 ( 9.38 ) | 4.73 ( 1.56 ) | 2.24 ( 0.58 ) |
| Hip arthroplasty | 3 | 3.78 ( 1.08 - 13.25 ) | 3.77 ( 4.97 ) | 3.25 ( 1.14 ) | 1.7 ( 0.1 ) |
| Mycobacterium avium complex infection | 3 | 8.18 ( 2.05 - 32.71 ) | 8.18 ( 12.6 ) | 5.79 ( 1.81 ) | 2.53 ( 0.83 ) |
| Anxiety disorder | 3 | 4.91 ( 1.35 - 17.83 ) | 4.91 ( 7.18 ) | 4.01 ( 1.36 ) | 2 ( 0.37 ) |
| Csf white blood cell count decreased | 3 | 49.08 ( 5.1 - 471.87 ) | 49.07 ( 35.32 ) | 13.02 ( 1.96 ) | 3.7 ( 1.77 ) |
| Mucocutaneous leishmaniasis | 3 | 16.36 ( 3.3 - 81.06 ) | 16.36 ( 21.63 ) | 8.68 ( 2.27 ) | 3.12 ( 1.31 ) |
| Glucocorticoid deficiency | 3 | 8.18 ( 2.05 - 32.71 ) | 8.18 ( 12.6 ) | 5.79 ( 1.81 ) | 2.53 ( 0.83 ) |
| Palmoplantar keratoderma | 3 | 16.36 ( 3.3 - 81.06 ) | 16.36 ( 21.63 ) | 8.68 ( 2.27 ) | 3.12 ( 1.31 ) |
| Bicytopenia | 3 | 4.46 ( 1.24 - 15.99 ) | 4.46 ( 6.33 ) | 3.72 ( 1.28 ) | 1.9 ( 0.27 ) |
| Right ventricular hypertrophy | 3 | 12.27 ( 2.75 - 54.83 ) | 12.27 ( 17.74 ) | 7.44 ( 2.13 ) | 2.9 ( 1.13 ) |
| Renal arteriosclerosis | 3 | 9.82 ( 2.35 - 41.08 ) | 9.81 ( 14.84 ) | 6.51 ( 1.96 ) | 2.7 ( 0.97 ) |
| Vitreous haemorrhage | 3 | 7.01 ( 1.81 - 27.12 ) | 7.01 ( 10.82 ) | 5.21 ( 1.68 ) | 2.38 ( 0.7 ) |
| Autoimmune dermatitis | 3 | 4.46 ( 1.24 - 15.99 ) | 4.46 ( 6.33 ) | 3.72 ( 1.28 ) | 1.9 ( 0.27 ) |
| Epstein-barr virus infection | 3 | 24.54 ( 4.1 - 146.88 ) | 24.53 ( 27.09 ) | 10.41 ( 2.33 ) | 3.38 ( 1.52 ) |
| Cushing's syndrome | 3 | 9.82 ( 2.35 - 41.08 ) | 9.81 ( 14.84 ) | 6.51 ( 1.96 ) | 2.7 ( 0.97 ) |
| Cutaneous vasculitis | 3 | 4.09 ( 1.15 - 14.49 ) | 4.09 ( 5.6 ) | 3.47 ( 1.2 ) | 1.8 ( 0.18 ) |
| Autoimmune cholangitis | 3 | 6.13 ( 1.63 - 23.13 ) | 6.13 ( 9.38 ) | 4.73 ( 1.56 ) | 2.24 ( 0.58 ) |
| Fungal oesophagitis | 3 | 8.18 ( 2.05 - 32.71 ) | 8.18 ( 12.6 ) | 5.79 ( 1.81 ) | 2.53 ( 0.83 ) |
| Graves' disease | 3 | 49.08 ( 5.1 - 471.87 ) | 49.07 ( 35.32 ) | 13.02 ( 1.96 ) | 3.7 ( 1.77 ) |
| Cortisol increased | 3 | 24.54 ( 4.1 - 146.88 ) | 24.53 ( 27.09 ) | 10.41 ( 2.33 ) | 3.38 ( 1.52 ) |
| Tertiary adrenal insufficiency | 3 | 16.36 ( 3.3 - 81.06 ) | 16.36 ( 21.63 ) | 8.68 ( 2.27 ) | 3.12 ( 1.31 ) |
| Infective exacerbation of chronic obstructive airways disease | 3 | 4.91 ( 1.35 - 17.83 ) | 4.91 ( 7.18 ) | 4.01 ( 1.36 ) | 2 ( 0.37 ) |
| Autoimmune myositis | 3 | 7.01 ( 1.81 - 27.12 ) | 7.01 ( 10.82 ) | 5.21 ( 1.68 ) | 2.38 ( 0.7 ) |
| Livedo reticularis | 3 | 3.51 ( 1.01 - 12.2 ) | 3.5 ( 4.42 ) | 3.06 ( 1.08 ) | 1.61 ( 0.02 ) |
| Trisomy 8 | 3 | 49.08 ( 5.1 - 471.87 ) | 49.07 ( 35.32 ) | 13.02 ( 1.96 ) | 3.7 ( 1.77 ) |
| Caesarean section | 3 | 49.08 ( 5.1 - 471.87 ) | 49.07 ( 35.32 ) | 13.02 ( 1.96 ) | 3.7 ( 1.77 ) |

Abbreviation: ROR, reporting odds ratio; PRR, proportional reporting ratio; EBGM, empirical Bayesian geometric mean; EBGM05, the lower limit of the 95% CI of EBGM; IC, information component; IC025, the lower limit of the 95% CI of the IC; CI, confidence interval; PT,preferred term.

Supplementary Table 4:

Top 50 most frequent adverse events for Carboplatin at the preferred term (PT) level in males from FAERS data

| PT | Case numbers | ROR(95%Cl) | PRR(χ2) | EBGM(EBGM05) | IC(IC025) |
| --- | --- | --- | --- | --- | --- |
| Anaemia* | 196 | 2.71 ( 2.32 - 3.16 ) | 2.66 ( 176.37 ) | 2.42 ( 2.13 ) | 1.28 ( 1.06 ) |
| Diarrhoea | 156 | 1.07 ( 0.91 - 1.27 ) | 1.07 ( 0.74 ) | 1.07 ( 0.93 ) | 0.1 ( -0.14 ) |
| Pneumonia* | 155 | 1.19 ( 1.01 - 1.41 ) | 1.19 ( 4.37 ) | 1.17 ( 1.02 ) | 0.23 ( -0.01 ) |
| Thrombocytopenia* | 139 | 3.08 ( 2.57 - 3.7 ) | 3.04 ( 161.02 ) | 2.71 ( 2.33 ) | 1.44 ( 1.18 ) |
| Neutropenia* | 128 | 2.59 ( 2.15 - 3.13 ) | 2.56 ( 105.97 ) | 2.35 ( 2.01 ) | 1.23 ( 0.96 ) |
| Nausea* | 121 | 1.47 ( 1.22 - 1.78 ) | 1.47 ( 16.62 ) | 1.43 ( 1.22 ) | 0.51 ( 0.24 ) |
| Leukopenia* | 121 | 6.23 ( 5.04 - 7.69 ) | 6.14 ( 376.96 ) | 4.71 ( 3.95 ) | 2.24 ( 1.94 ) |
| Fatigue* | 115 | 1.32 ( 1.09 - 1.6 ) | 1.32 ( 8.17 ) | 1.29 ( 1.1 ) | 0.37 ( 0.09 ) |
| Pyrexia | 112 | 1.05 ( 0.87 - 1.28 ) | 1.05 ( 0.27 ) | 1.05 ( 0.89 ) | 0.07 ( -0.21 ) |
| Pancytopenia* | 106 | 4.98 ( 4 - 6.2 ) | 4.92 ( 253.97 ) | 4 ( 3.33 ) | 2 ( 1.69 ) |
| Febrile neutropenia* | 105 | 2 ( 1.63 - 2.45 ) | 1.98 ( 45.72 ) | 1.87 ( 1.58 ) | 0.9 ( 0.61 ) |
| Dyspnoea | 96 | 0.82 ( 0.66 - 1 ) | 0.82 ( 3.77 ) | 0.83 ( 0.7 ) | -0.27 ( -0.58 ) |
| General physical health deterioration* | 96 | 1.95 ( 1.58 - 2.42 ) | 1.94 ( 39.27 ) | 1.84 ( 1.54 ) | 0.88 ( 0.57 ) |
| Vomiting* | 90 | 1.45 ( 1.17 - 1.8 ) | 1.44 ( 11.31 ) | 1.41 ( 1.17 ) | 0.49 ( 0.18 ) |
| Dehydration* | 77 | 1.5 ( 1.19 - 1.9 ) | 1.49 ( 11.57 ) | 1.45 ( 1.19 ) | 0.54 ( 0.2 ) |
| Acute kidney injury* | 64 | 1.98 ( 1.53 - 2.57 ) | 1.97 ( 27.5 ) | 1.87 ( 1.5 ) | 0.9 ( 0.52 ) |
| Constipation* | 59 | 1.75 ( 1.34 - 2.3 ) | 1.75 ( 17.12 ) | 1.67 ( 1.34 ) | 0.74 ( 0.35 ) |
| Sepsis* | 57 | 1.51 ( 1.15 - 1.98 ) | 1.5 ( 8.85 ) | 1.46 ( 1.16 ) | 0.55 ( 0.15 ) |
| Asthenia | 56 | 0.81 ( 0.62 - 1.07 ) | 0.82 ( 2.24 ) | 0.82 ( 0.66 ) | -0.28 ( -0.67 ) |
| Interstitial lung disease | 56 | 0.57 ( 0.43 - 0.74 ) | 0.57 ( 17.77 ) | 0.58 ( 0.47 ) | -0.77 ( -1.16 ) |
| Mucosal inflammation* | 50 | 3.26 ( 2.4 - 4.42 ) | 3.24 ( 64.55 ) | 2.86 ( 2.22 ) | 1.52 ( 1.08 ) |
| Rash | 50 | 0.5 ( 0.37 - 0.66 ) | 0.5 ( 24.72 ) | 0.51 ( 0.41 ) | -0.96 ( -1.37 ) |
| Pneumonitis | 48 | 0.65 ( 0.48 - 0.87 ) | 0.65 ( 8.77 ) | 0.66 ( 0.52 ) | -0.59 ( -1.01 ) |
| Hypotension* | 48 | 1.6 ( 1.19 - 2.15 ) | 1.6 ( 9.73 ) | 1.54 ( 1.2 ) | 0.62 ( 0.19 ) |
| Platelet count decreased | 48 | 1.2 ( 0.89 - 1.61 ) | 1.2 ( 1.48 ) | 1.19 ( 0.93 ) | 0.24 ( -0.18 ) |
| Pulmonary embolism | 47 | 1.07 ( 0.8 - 1.44 ) | 1.07 ( 0.21 ) | 1.07 ( 0.83 ) | 0.09 ( -0.34 ) |
| Respiratory failure | 45 | 0.81 ( 0.6 - 1.09 ) | 0.81 ( 1.91 ) | 0.82 ( 0.64 ) | -0.29 ( -0.72 ) |
| Polyneuropathy* | 44 | 9.76 ( 6.7 - 14.21 ) | 9.71 ( 213.55 ) | 6.41 ( 4.68 ) | 2.68 ( 2.17 ) |
| Infection* | 43 | 2.35 ( 1.7 - 3.24 ) | 2.34 ( 28.83 ) | 2.17 ( 1.66 ) | 1.12 ( 0.65 ) |
| Stomatitis* | 41 | 1.88 ( 1.36 - 2.6 ) | 1.87 ( 14.94 ) | 1.78 ( 1.36 ) | 0.83 ( 0.36 ) |
| Cough | 40 | 1 ( 0.72 - 1.37 ) | 1 ( 0 ) | 1 ( 0.76 ) | 0 ( -0.47 ) |
| White blood cell count decreased | 40 | 1.38 ( 1 - 1.91 ) | 1.38 ( 3.89 ) | 1.35 ( 1.03 ) | 0.43 ( -0.04 ) |
| Decreased appetite | 39 | 0.52 ( 0.38 - 0.72 ) | 0.52 ( 16.69 ) | 0.54 ( 0.41 ) | -0.9 ( -1.36 ) |
| Atrial fibrillation* | 36 | 1.47 ( 1.04 - 2.06 ) | 1.46 ( 4.84 ) | 1.42 ( 1.07 ) | 0.51 ( 0.01 ) |
| Hypothyroidism* | 36 | 1.58 ( 1.12 - 2.22 ) | 1.58 ( 6.91 ) | 1.52 ( 1.14 ) | 0.61 ( 0.11 ) |
| Neutrophil count decreased | 35 | 1.22 ( 0.86 - 1.71 ) | 1.21 ( 1.23 ) | 1.2 ( 0.9 ) | 0.26 ( -0.24 ) |
| Oedema peripheral | 35 | 1.16 ( 0.82 - 1.63 ) | 1.16 ( 0.69 ) | 1.15 ( 0.86 ) | 0.2 ( -0.3 ) |
| Haemoptysis | 34 | 0.89 ( 0.63 - 1.26 ) | 0.89 ( 0.42 ) | 0.9 ( 0.67 ) | -0.15 ( -0.66 ) |
| Dysphagia* | 34 | 1.69 ( 1.19 - 2.41 ) | 1.69 ( 8.64 ) | 1.62 ( 1.21 ) | 0.7 ( 0.19 ) |
| Neutropenic sepsis* | 33 | 4.54 ( 3.08 - 6.68 ) | 4.52 ( 70.5 ) | 3.74 ( 2.7 ) | 1.9 ( 1.35 ) |
| Hypokalaemia* | 33 | 2.56 ( 1.77 - 3.71 ) | 2.56 ( 27.01 ) | 2.34 ( 1.72 ) | 1.23 ( 0.7 ) |
| Pruritus | 33 | 1.31 ( 0.91 - 1.86 ) | 1.3 ( 2.17 ) | 1.28 ( 0.95 ) | 0.36 ( -0.16 ) |
| Haemoglobin decreased | 32 | 1.24 ( 0.87 - 1.78 ) | 1.24 ( 1.38 ) | 1.22 ( 0.9 ) | 0.29 ( -0.23 ) |
| Alopecia* | 32 | 4.22 ( 2.85 - 6.23 ) | 4.2 ( 61.82 ) | 3.53 ( 2.55 ) | 1.82 ( 1.27 ) |
| Renal failure | 32 | 1.2 ( 0.84 - 1.72 ) | 1.2 ( 0.99 ) | 1.19 ( 0.88 ) | 0.24 ( -0.28 ) |
| Pleural effusion | 31 | 0.52 ( 0.36 - 0.74 ) | 0.52 ( 13.35 ) | 0.54 ( 0.4 ) | -0.9 ( -1.42 ) |
| Abdominal pain | 31 | 1.33 ( 0.92 - 1.92 ) | 1.33 ( 2.32 ) | 1.3 ( 0.96 ) | 0.38 ( -0.15 ) |
| Malaise | 30 | 0.86 ( 0.6 - 1.25 ) | 0.87 ( 0.6 ) | 0.87 ( 0.64 ) | -0.2 ( -0.73 ) |
| Chest pain | 29 | 1.28 ( 0.87 - 1.87 ) | 1.28 ( 1.61 ) | 1.26 ( 0.91 ) | 0.33 ( -0.22 ) |
| Arthralgia* | 27 | 1.07 ( 0.73 - 1.59 ) | 1.07 ( 0.12 ) | 1.07 ( 0.77 ) | 0.09 ( -0.47 ) |

Abbreviation: Asterisks (*) indicate statistically significant signals in algorithm; ROR, reporting odds ratio; PRR, proportional reporting ratio; EBGM, empirical Bayesian geometric mean; EBGM05, the lower limit of the 95% CI of EBGM; IC, information component; IC025, the lower limit of the 95% CI of the IC; CI, confidence interval; PT,preferred term; AEs, adverse events.

Supplementary Table 5:

Top 50 most frequent adverse events for Carboplatin at the PT level in females from FAERS data

| PT | Case numbers | ROR(95%Cl) | PRR(χ2) | EBGM(EBGM05) | IC(IC025) |
| --- | --- | --- | --- | --- | --- |
| Nausea* | 146 | 1.42 ( 1.2 - 1.68 ) | 1.41 ( 16.26 ) | 1.38 ( 1.19 ) | 0.46 ( 0.21 ) |
| Anaemia* | 145 | 2.82 ( 2.36 - 3.36 ) | 2.77 ( 143.37 ) | 2.53 ( 2.18 ) | 1.34 ( 1.08 ) |
| Diarrhoea | 122 | 0.73 ( 0.61 - 0.88 ) | 0.74 ( 11.28 ) | 0.75 ( 0.64 ) | -0.42 ( -0.69 ) |
| Fatigue* | 110 | 1.36 ( 1.12 - 1.65 ) | 1.35 ( 9.45 ) | 1.33 ( 1.13 ) | 0.41 ( 0.12 ) |
| Vomiting | 88 | 1.13 ( 0.91 - 1.41 ) | 1.13 ( 1.28 ) | 1.12 ( 0.94 ) | 0.17 ( -0.15 ) |
| Pneumonia* | 88 | 1.48 ( 1.19 - 1.84 ) | 1.47 ( 12.34 ) | 1.43 ( 1.19 ) | 0.52 ( 0.2 ) |
| Dyspnoea | 78 | 0.99 ( 0.79 - 1.24 ) | 0.99 ( 0.01 ) | 0.99 ( 0.82 ) | -0.01 ( -0.35 ) |
| Thrombocytopenia* | 73 | 2.78 ( 2.17 - 3.57 ) | 2.76 ( 71.28 ) | 2.52 ( 2.05 ) | 1.34 ( 0.98 ) |
| Febrile neutropenia* | 64 | 2.77 ( 2.12 - 3.6 ) | 2.75 ( 61.83 ) | 2.51 ( 2.01 ) | 1.33 ( 0.95 ) |
| Asthenia | 62 | 1.15 ( 0.89 - 1.49 ) | 1.15 ( 1.17 ) | 1.14 ( 0.92 ) | 0.19 ( -0.18 ) |
| Constipation* | 62 | 1.72 ( 1.32 - 2.23 ) | 1.71 ( 16.84 ) | 1.65 ( 1.32 ) | 0.72 ( 0.34 ) |
| General physical health deterioration* | 60 | 2.04 ( 1.56 - 2.67 ) | 2.03 ( 28.34 ) | 1.93 ( 1.54 ) | 0.95 ( 0.56 ) |
| Neutropenia* | 60 | 2.2 ( 1.68 - 2.88 ) | 2.18 ( 34.51 ) | 2.06 ( 1.64 ) | 1.04 ( 0.65 ) |
| Pancytopenia* | 55 | 4.31 ( 3.21 - 5.79 ) | 4.28 ( 111.62 ) | 3.64 ( 2.84 ) | 1.86 ( 1.44 ) |
| Leukopenia* | 54 | 4.46 ( 3.31 - 6.02 ) | 4.43 ( 115.16 ) | 3.75 ( 2.92 ) | 1.91 ( 1.48 ) |
| Decreased appetite | 54 | 0.91 ( 0.69 - 1.19 ) | 0.91 ( 0.49 ) | 0.91 ( 0.72 ) | -0.13 ( -0.53 ) |
| Pulmonary embolism* | 50 | 1.48 ( 1.11 - 1.97 ) | 1.47 ( 7.08 ) | 1.44 ( 1.13 ) | 0.52 ( 0.1 ) |
| Abdominal pain* | 49 | 1.85 ( 1.38 - 2.49 ) | 1.84 ( 17.25 ) | 1.77 ( 1.38 ) | 0.82 ( 0.39 ) |
| Acute kidney injury* | 49 | 2.57 ( 1.9 - 3.47 ) | 2.56 ( 40.82 ) | 2.36 ( 1.84 ) | 1.24 ( 0.81 ) |
| Pyrexia | 46 | 0.74 ( 0.55 - 1 ) | 0.75 ( 3.84 ) | 0.76 ( 0.59 ) | -0.4 ( -0.83 ) |
| Dehydration | 45 | 1.01 ( 0.75 - 1.36 ) | 1.01 ( 0 ) | 1.01 ( 0.78 ) | 0.01 ( -0.43 ) |
| Alopecia* | 44 | 1.74 ( 1.27 - 2.37 ) | 1.73 ( 12.5 ) | 1.67 ( 1.29 ) | 0.74 ( 0.29 ) |
| Platelet count decreased* | 42 | 1.78 ( 1.29 - 2.44 ) | 1.77 ( 12.91 ) | 1.7 ( 1.3 ) | 0.77 ( 0.31 ) |
| Hepatitis* | 42 | 4.59 ( 3.26 - 6.44 ) | 4.56 ( 93.11 ) | 3.83 ( 2.88 ) | 1.94 ( 1.45 ) |
| Pneumonitis | 41 | 1.13 ( 0.82 - 1.55 ) | 1.12 ( 0.54 ) | 1.12 ( 0.86 ) | 0.16 ( -0.3 ) |
| Neoplasm progression | 38 | 1.33 ( 0.95 - 1.85 ) | 1.32 ( 2.82 ) | 1.3 ( 0.99 ) | 0.38 ( -0.1 ) |
| Neuropathy peripheral* | 38 | 2.86 ( 2.03 - 4.03 ) | 2.84 ( 39.25 ) | 2.59 ( 1.94 ) | 1.37 ( 0.88 ) |
| Mucosal inflammation* | 38 | 2.93 ( 2.08 - 4.13 ) | 2.92 ( 41.22 ) | 2.65 ( 1.98 ) | 1.4 ( 0.91 ) |
| Hypokalaemia** | 37 | 1.99 ( 1.42 - 2.8 ) | 1.98 ( 16.31 ) | 1.89 ( 1.42 ) | 0.92 ( 0.42 ) |
| White blood cell count decreased | 34 | 1.65 ( 1.16 - 2.35 ) | 1.65 ( 7.9 ) | 1.59 ( 1.18 ) | 0.67 ( 0.16 ) |
| Sepsis* | 31 | 1.65 ( 1.14 - 2.38 ) | 1.64 ( 7.16 ) | 1.59 ( 1.17 ) | 0.67 ( 0.13 ) |
| Rash | 31 | 0.32 ( 0.22 - 0.45 ) | 0.32 ( 44.53 ) | 0.33 ( 0.25 ) | -1.59 ( -2.1 ) |
| Dizziness | 31 | 1.11 ( 0.77 - 1.59 ) | 1.11 ( 0.3 ) | 1.1 ( 0.81 ) | 0.14 ( -0.39 ) |
| Cough | 29 | 0.79 ( 0.54 - 1.14 ) | 0.79 ( 1.59 ) | 0.8 ( 0.58 ) | -0.33 ( -0.87 ) |
| Headache | 29 | 1.02 ( 0.7 - 1.49 ) | 1.02 ( 0.01 ) | 1.02 ( 0.74 ) | 0.03 ( -0.51 ) |
| Chest pain* | 27 | 1.55 ( 1.05 - 2.3 ) | 1.55 ( 4.87 ) | 1.51 ( 1.08 ) | 0.59 ( 0.02 ) |
| Haemoglobin decreased* | 27 | 1.77 ( 1.19 - 2.63 ) | 1.77 ( 8.21 ) | 1.7 ( 1.22 ) | 0.76 ( 0.19 ) |
| Immune-mediated enterocolitis* | 27 | 5.81 ( 3.76 - 8.98 ) | 5.79 ( 80.86 ) | 4.62 ( 3.21 ) | 2.21 ( 1.6 ) |
| Dysphagia | 26 | 1.3 ( 0.87 - 1.94 ) | 1.3 ( 1.69 ) | 1.28 ( 0.92 ) | 0.36 ( -0.22 ) |
| Blood creatinine increased | 25 | 1.49 ( 0.99 - 2.24 ) | 1.49 ( 3.71 ) | 1.45 ( 1.03 ) | 0.54 ( -0.05 ) |
| Hepatic failure* | 24 | 5.23 ( 3.32 - 8.25 ) | 5.21 ( 63.25 ) | 4.26 ( 2.91 ) | 2.09 ( 1.45 ) |
| Infection* | 23 | 1.86 ( 1.21 - 2.85 ) | 1.85 ( 8.2 ) | 1.77 ( 1.24 ) | 0.83 ( 0.21 ) |
| Pleural effusion | 23 | 0.52 ( 0.34 - 0.79 ) | 0.52 ( 9.71 ) | 0.54 ( 0.38 ) | -0.89 ( -1.49 ) |
| Polyneuropathy* | 23 | 7.75 ( 4.75 - 12.66 ) | 7.73 ( 94 ) | 5.69 ( 3.78 ) | 2.51 ( 1.83 ) |
| Weight decreased | 23 | 0.7 ( 0.46 - 1.07 ) | 0.7 ( 2.78 ) | 0.71 ( 0.5 ) | -0.48 ( -1.09 ) |
| Hypotension | 22 | 1.29 ( 0.83 - 1.98 ) | 1.28 ( 1.3 ) | 1.27 ( 0.88 ) | 0.34 ( -0.28 ) |
| Pruritus | 22 | 0.85 ( 0.55 - 1.31 ) | 0.85 ( 0.55 ) | 0.86 ( 0.6 ) | -0.22 ( -0.84 ) |
| Hyponatraemia | 22 | 1.43 ( 0.93 - 2.21 ) | 1.43 ( 2.64 ) | 1.4 ( 0.97 ) | 0.48 ( -0.14 ) |
| Hypersensitivity* | 21 | 2.24 ( 1.42 - 3.53 ) | 2.24 ( 12.81 ) | 2.1 ( 1.44 ) | 1.07 ( 0.42 ) |
| Gamma-glutamyltransferase increased* | 21 | 2.27 ( 1.44 - 3.58 ) | 2.27 ( 13.2 ) | 2.12 ( 1.45 ) | 1.09 ( 0.43 ) |

Abbreviation: Asterisks (*) indicate statistically significant signals in algorithm; ROR, reporting odds ratio; PRR, proportional reporting ratio; EBGM, empirical Bayesian geometric mean; EBGM05, the lower limit of the 95% CI of EBGM; IC, information component; IC025, the lower limit of the 95% CI of the IC; CI, confidence interval; PT,preferred term; AEs, adverse events.

Supplementary Table 6:

Adverse events at the PT level for Carboplatin in patients aged under 18 from FAERS data

| PT | Case numbers | ROR(95%Cl) | PRR(χ2) | EBGM(EBGM05) | IC(IC025) |
| --- | --- | --- | --- | --- | --- |
| Anaemia | 2 | 3.57 ( 0.46 - 27.68 ) | 3.25 ( 1.66 ) | 2.12 ( 0.38 ) | 1.09 ( -1.04 ) |
| General physical health deterioration | 2 | Inf ( NaN - Inf ) | Inf ( 6.7 ) | 4.25 ( 0 ) | 2.09 ( -0.29 ) |
| Leukopenia | 2 | Inf ( NaN - Inf ) | Inf ( 6.7 ) | 4.25 ( 0 ) | 2.09 ( -0.29 ) |
| Candida infection | 1 | Inf ( NaN - Inf ) | Inf ( 3.3 ) | 4.25 ( 0 ) | 2.09 ( -0.81 ) |
| Decreased appetite | 1 | Inf ( NaN - Inf ) | Inf ( 3.3 ) | 4.25 ( 0 ) | 2.09 ( -0.81 ) |
| Polyneuropathy | 1 | Inf ( NaN - Inf ) | Inf ( 3.3 ) | 4.25 ( 0 ) | 2.09 ( -0.81 ) |
| Taste disorder | 1 | Inf ( NaN - Inf ) | Inf ( 3.3 ) | 4.25 ( 0 ) | 2.09 ( -0.81 ) |
| Dehydration | 1 | Inf ( NaN - Inf ) | Inf ( 3.3 ) | 4.25 ( 0 ) | 2.09 ( -0.81 ) |
| Metastases to central nervous system | 1 | 3.4 ( 0.2 - 57.67 ) | 3.25 ( 0.8 ) | 2.12 ( 0.2 ) | 1.09 ( -1.56 ) |
| Thrombocytopenia | 1 | Inf ( NaN - Inf ) | Inf ( 3.3 ) | 4.25 ( 0 ) | 2.09 ( -0.81 ) |
| Urosepsis | 1 | Inf ( NaN - Inf ) | Inf ( 3.3 ) | 4.25 ( 0 ) | 2.09 ( -0.81 ) |
| Non-small cell lung cancer | 1 | 3.4 ( 0.2 - 57.67 ) | 3.25 ( 0.8 ) | 2.12 ( 0.2 ) | 1.09 ( -1.56 ) |
| Nausea | 1 | 3.4 ( 0.2 - 57.67 ) | 3.25 ( 0.8 ) | 2.12 ( 0.2 ) | 1.09 ( -1.56 ) |

Abbreviation: Asterisks (*) indicate statistically significant signals in algorithm; ROR, reporting odds ratio; PRR, proportional reporting ratio; EBGM, empirical Bayesian geometric mean; EBGM05, the lower limit of the 95% CI of EBGM; IC, information component; IC025, the lower limit of the 95% CI of the IC; CI, confidence interval; PT, preferred term.

Supplementary Table 7:

Top 50 most frequent adverse events for Carboplatin at the PT level in patients aged 18 to 65 from FAERS data

| PT | Case numbers | ROR(95%Cl) | PRR(χ2) | EBGM(EBGM05) | IC(IC025) |
| --- | --- | --- | --- | --- | --- |
| Nausea* | 127 | 1.47 ( 1.22 - 1.77 ) | 1.46 ( 16.89 ) | 1.42 ( 1.21 ) | 0.5 ( 0.23 ) |
| Anaemia* | 117 | 2.22 ( 1.82 - 2.7 ) | 2.19 ( 66.56 ) | 2.04 ( 1.73 ) | 1.03 ( 0.74 ) |
| Pneumonia* | 97 | 1.34 ( 1.09 - 1.66 ) | 1.34 ( 7.7 ) | 1.31 ( 1.1 ) | 0.39 ( 0.08 ) |
| Diarrhoea | 90 | 0.79 ( 0.64 - 0.98 ) | 0.8 ( 4.48 ) | 0.81 ( 0.68 ) | -0.31 ( -0.62 ) |
| Fatigue* | 86 | 1.38 ( 1.11 - 1.73 ) | 1.38 ( 8.26 ) | 1.35 ( 1.12 ) | 0.43 ( 0.1 ) |
| Thrombocytopenia* | 80 | 2.52 ( 1.98 - 3.2 ) | 2.5 ( 61.62 ) | 2.28 ( 1.86 ) | 1.19 ( 0.84 ) |
| Vomiting | 79 | 1.14 ( 0.91 - 1.44 ) | 1.14 ( 1.25 ) | 1.13 ( 0.93 ) | 0.17 ( -0.16 ) |
| Dyspnoea | 75 | 0.95 ( 0.75 - 1.2 ) | 0.95 ( 0.17 ) | 0.96 ( 0.78 ) | -0.07 ( -0.41 ) |
| Febrile neutropenia* | 75 | 2.44 ( 1.91 - 3.12 ) | 2.42 ( 54.06 ) | 2.22 ( 1.81 ) | 1.15 ( 0.79 ) |
| Pyrexia | 68 | 0.93 ( 0.73 - 1.19 ) | 0.93 ( 0.32 ) | 0.94 ( 0.76 ) | -0.1 ( -0.46 ) |
| Leukopenia* | 65 | 4.43 ( 3.35 - 5.85 ) | 4.38 ( 130.95 ) | 3.6 ( 2.85 ) | 1.85 ( 1.45 ) |
| Neutropenia* | 61 | 1.82 ( 1.39 - 2.37 ) | 1.81 ( 19.66 ) | 1.72 ( 1.37 ) | 0.78 ( 0.39 ) |
| General physical health deterioration* | 55 | 1.67 ( 1.26 - 2.21 ) | 1.66 ( 13.11 ) | 1.59 ( 1.26 ) | 0.67 ( 0.27 ) |
| Cough* | 52 | 1.51 ( 1.13 - 2.01 ) | 1.5 ( 7.99 ) | 1.46 ( 1.15 ) | 0.54 ( 0.13 ) |
| Pulmonary embolism | 51 | 1.32 ( 0.99 - 1.75 ) | 1.31 ( 3.51 ) | 1.29 ( 1.01 ) | 0.36 ( -0.05 ) |
| Pancytopenia* | 46 | 3.83 ( 2.77 - 5.31 ) | 3.81 ( 75.7 ) | 3.23 ( 2.46 ) | 1.69 ( 1.23 ) |
| Dehydration | 39 | 1.15 ( 0.83 - 1.6 ) | 1.15 ( 0.71 ) | 1.14 ( 0.87 ) | 0.19 ( -0.29 ) |
| Acute kidney injury* | 39 | 1.95 ( 1.4 - 2.73 ) | 1.95 ( 15.88 ) | 1.83 ( 1.39 ) | 0.88 ( 0.39 ) |
| Pneumonitis | 37 | 0.9 ( 0.65 - 1.26 ) | 0.9 ( 0.38 ) | 0.91 ( 0.69 ) | -0.14 ( -0.62 ) |
| Platelet count decreased* | 37 | 1.5 ( 1.07 - 2.11 ) | 1.5 ( 5.57 ) | 1.45 ( 1.09 ) | 0.54 ( 0.05 ) |
| Asthenia | 35 | 0.83 ( 0.59 - 1.17 ) | 0.83 ( 1.16 ) | 0.84 ( 0.63 ) | -0.25 ( -0.75 ) |
| White blood cell count decreased* | 35 | 1.61 ( 1.14 - 2.29 ) | 1.61 ( 7.28 ) | 1.55 ( 1.15 ) | 0.63 ( 0.12 ) |
| Mucosal inflammation* | 33 | 2.73 ( 1.88 - 3.96 ) | 2.72 ( 30.23 ) | 2.45 ( 1.79 ) | 1.29 ( 0.76 ) |
| Rash | 33 | 0.47 ( 0.34 - 0.67 ) | 0.48 ( 18.41 ) | 0.49 ( 0.37 ) | -1.01 ( -1.52 ) |
| Constipation | 32 | 1.28 ( 0.89 - 1.84 ) | 1.28 ( 1.78 ) | 1.25 ( 0.93 ) | 0.33 ( -0.2 ) |
| Haemoglobin decreased* | 31 | 1.56 ( 1.07 - 2.25 ) | 1.55 ( 5.52 ) | 1.5 ( 1.1 ) | 0.58 ( 0.05 ) |
| Neuropathy peripheral* | 30 | 2.51 ( 1.7 - 3.7 ) | 2.5 ( 23.05 ) | 2.28 ( 1.65 ) | 1.19 ( 0.63 ) |
| Sepsis | 30 | 1.37 ( 0.94 - 1.99 ) | 1.37 ( 2.7 ) | 1.33 ( 0.97 ) | 0.42 ( -0.13 ) |
| Dizziness | 28 | 1.27 ( 0.86 - 1.87 ) | 1.27 ( 1.45 ) | 1.25 ( 0.9 ) | 0.32 ( -0.24 ) |
| Decreased appetite | 28 | 0.65 ( 0.45 - 0.95 ) | 0.65 ( 4.96 ) | 0.67 ( 0.49 ) | -0.58 ( -1.13 ) |
| Respiratory failure | 27 | 0.88 ( 0.59 - 1.3 ) | 0.88 ( 0.43 ) | 0.88 ( 0.64 ) | -0.18 ( -0.74 ) |
| Hepatitis* | 27 | 3.26 ( 2.15 - 4.96 ) | 3.25 ( 34.46 ) | 2.84 ( 2 ) | 1.51 ( 0.91 ) |
| Neutropenic sepsis* | 26 | 5.14 ( 3.28 - 8.04 ) | 5.12 ( 63.83 ) | 4.05 ( 2.78 ) | 2.02 ( 1.39 ) |
| Pruritus | 25 | 1.44 ( 0.95 - 2.17 ) | 1.43 ( 3 ) | 1.39 ( 0.99 ) | 0.48 ( -0.11 ) |
| Infection* | 25 | 1.96 ( 1.29 - 2.98 ) | 1.96 ( 10.36 ) | 1.84 ( 1.3 ) | 0.88 ( 0.28 ) |
| Hypothyroidism* | 25 | 1.82 ( 1.2 - 2.75 ) | 1.81 ( 8.11 ) | 1.72 ( 1.22 ) | 0.78 ( 0.19 ) |
| Abdominal pain | 24 | 1.02 ( 0.68 - 1.55 ) | 1.02 ( 0.01 ) | 1.02 ( 0.72 ) | 0.03 ( -0.57 ) |
| Chest pain | 24 | 1.13 ( 0.75 - 1.72 ) | 1.13 ( 0.34 ) | 1.12 ( 0.79 ) | 0.17 ( -0.43 ) |
| Pleural effusion | 24 | 0.59 ( 0.39 - 0.88 ) | 0.59 ( 6.73 ) | 0.6 ( 0.43 ) | -0.73 ( -1.32 ) |
| Malaise | 23 | 1.02 ( 0.67 - 1.56 ) | 1.02 ( 0.01 ) | 1.02 ( 0.72 ) | 0.03 ( -0.58 ) |
| Hypokalaemia* | 23 | 1.65 ( 1.07 - 2.55 ) | 1.65 ( 5.31 ) | 1.58 ( 1.1 ) | 0.66 ( 0.04 ) |
| Headache | 23 | 0.85 ( 0.56 - 1.3 ) | 0.85 ( 0.58 ) | 0.86 ( 0.6 ) | -0.22 ( -0.83 ) |
| Alopecia* | 23 | 1.58 ( 1.02 - 2.42 ) | 1.57 ( 4.35 ) | 1.52 ( 1.06 ) | 0.6 ( -0.02 ) |
| Pain | 22 | 0.81 ( 0.53 - 1.25 ) | 0.82 ( 0.87 ) | 0.83 ( 0.58 ) | -0.28 ( -0.9 ) |
| Bone pain* | 22 | 3.46 ( 2.17 - 5.51 ) | 3.45 ( 30.93 ) | 2.98 ( 2.02 ) | 1.57 ( 0.91 ) |
| Abdominal pain upper* | 22 | 2.33 ( 1.48 - 3.65 ) | 2.32 ( 14.31 ) | 2.14 ( 1.47 ) | 1.1 ( 0.45 ) |
| Dysphagia | 21 | 1.36 ( 0.87 - 2.12 ) | 1.35 ( 1.78 ) | 1.32 ( 0.91 ) | 0.4 ( -0.24 ) |
| Arthralgia | 20 | 0.97 ( 0.62 - 1.53 ) | 0.97 ( 0.01 ) | 0.98 ( 0.67 ) | -0.04 ( -0.69 ) |
| Stomatitis | 20 | 1.06 ( 0.67 - 1.68 ) | 1.06 ( 0.07 ) | 1.06 ( 0.72 ) | 0.08 ( -0.57 ) |
| Oedema peripheral | 20 | 1.09 ( 0.69 - 1.72 ) | 1.09 ( 0.14 ) | 1.08 ( 0.74 ) | 0.12 ( -0.54 ) |

Abbreviation: Asterisks (*) indicate statistically significant signals in algorithm; ROR, reporting odds ratio; PRR, proportional reporting ratio; EBGM, empirical Bayesian geometric mean; EBGM05, the lower limit of the 95% CI of EBGM; IC, information component; IC025, the lower limit of the 95% CI of the IC; CI, confidence interval; PT, preferred term.

Supplementary Table 8:

Top 50 most frequent adverse events for Carboplatin at the PT level in patients aged over 65 from FAERS data

| PT | Case numbers | ROR(95%Cl) | PRR(χ2) | EBGM(EBGM05) | IC(IC025) |
| --- | --- | --- | --- | --- | --- |
| Anaemia* | 187 | 2.6 ( 2.22 - 3.04 ) | 2.56 ( 152.56 ) | 2.32 ( 2.04 ) | 1.22 ( 0.99 ) |
| Diarrhoea | 164 | 0.9 ( 0.77 - 1.06 ) | 0.91 ( 1.55 ) | 0.91 ( 0.8 ) | -0.13 ( -0.37 ) |
| Pneumonia* | 134 | 1.25 ( 1.05 - 1.5 ) | 1.25 ( 6.13 ) | 1.23 ( 1.06 ) | 0.3 ( 0.04 ) |
| Nausea* | 127 | 1.43 ( 1.19 - 1.72 ) | 1.42 ( 14.84 ) | 1.39 ( 1.19 ) | 0.47 ( 0.2 ) |
| Fatigue* | 124 | 1.5 ( 1.25 - 1.81 ) | 1.5 ( 18.68 ) | 1.45 ( 1.24 ) | 0.54 ( 0.26 ) |
| Neutropenia* | 121 | 3.02 ( 2.48 - 3.67 ) | 2.98 ( 133.28 ) | 2.65 ( 2.24 ) | 1.4 ( 1.12 ) |
| Thrombocytopenia* | 114 | 3.18 ( 2.59 - 3.9 ) | 3.14 ( 137.91 ) | 2.76 ( 2.33 ) | 1.47 ( 1.17 ) |
| Pancytopenia* | 100 | 5.04 ( 4.01 - 6.33 ) | 4.98 ( 238.12 ) | 3.97 ( 3.28 ) | 1.99 ( 1.67 ) |
| Leukopenia* | 89 | 5.53 ( 4.33 - 7.07 ) | 5.47 ( 237.56 ) | 4.26 ( 3.47 ) | 2.09 ( 1.74 ) |
| Dyspnoea | 88 | 0.86 ( 0.7 - 1.07 ) | 0.87 ( 1.74 ) | 0.87 ( 0.73 ) | -0.19 ( -0.51 ) |
| Vomiting* | 87 | 1.3 ( 1.04 - 1.62 ) | 1.29 ( 5.35 ) | 1.27 ( 1.06 ) | 0.34 ( 0.02 ) |
| General physical health deterioration* | 86 | 2.06 ( 1.64 - 2.58 ) | 2.04 ( 40.48 ) | 1.92 ( 1.58 ) | 0.94 ( 0.61 ) |
| Constipation* | 86 | 2.07 ( 1.65 - 2.6 ) | 2.06 ( 41.4 ) | 1.93 ( 1.6 ) | 0.95 ( 0.62 ) |
| Febrile neutropenia* | 84 | 1.92 ( 1.53 - 2.41 ) | 1.91 ( 32.24 ) | 1.8 ( 1.49 ) | 0.85 ( 0.52 ) |
| Pyrexia | 82 | 0.92 ( 0.74 - 1.15 ) | 0.92 ( 0.52 ) | 0.93 ( 0.77 ) | -0.11 ( -0.44 ) |
| Dehydration* | 79 | 1.31 ( 1.04 - 1.66 ) | 1.31 ( 5.4 ) | 1.29 ( 1.06 ) | 0.36 ( 0.03 ) |
| Asthenia | 75 | 1.08 ( 0.85 - 1.37 ) | 1.08 ( 0.41 ) | 1.07 ( 0.88 ) | 0.1 ( -0.24 ) |
| Acute kidney injury* | 73 | 2.12 ( 1.66 - 2.71 ) | 2.11 ( 37.34 ) | 1.97 ( 1.6 ) | 0.98 ( 0.62 ) |
| Decreased appetite | 59 | 0.7 ( 0.54 - 0.91 ) | 0.7 ( 7.17 ) | 0.72 ( 0.58 ) | -0.48 ( -0.86 ) |
| Abdominal pain* | 53 | 1.98 ( 1.48 - 2.64 ) | 1.97 ( 22.37 ) | 1.85 ( 1.46 ) | 0.89 ( 0.47 ) |
| Pneumonitis | 49 | 0.85 ( 0.63 - 1.13 ) | 0.85 ( 1.26 ) | 0.86 ( 0.67 ) | -0.22 ( -0.64 ) |
| Alopecia* | 49 | 3.08 ( 2.26 - 4.19 ) | 3.06 ( 56.52 ) | 2.71 ( 2.09 ) | 1.44 ( 0.99 ) |
| Platelet count decreased | 48 | 1.32 ( 0.98 - 1.77 ) | 1.32 ( 3.35 ) | 1.29 ( 1.01 ) | 0.37 ( -0.06 ) |
| Sepsis* | 48 | 1.49 ( 1.11 - 2.01 ) | 1.49 ( 7.02 ) | 1.44 ( 1.12 ) | 0.53 ( 0.1 ) |
| Hypokalaemia* | 47 | 2.37 ( 1.74 - 3.22 ) | 2.36 ( 31.69 ) | 2.17 ( 1.67 ) | 1.12 ( 0.67 ) |
| Hypotension* | 46 | 1.67 ( 1.23 - 2.27 ) | 1.67 ( 11.06 ) | 1.6 ( 1.24 ) | 0.68 ( 0.23 ) |
| Mucosal inflammation* | 45 | 3.06 ( 2.22 - 4.23 ) | 3.05 ( 51.42 ) | 2.7 ( 2.06 ) | 1.43 ( 0.97 ) |
| Polyneuropathy* | 42 | 8.71 ( 5.94 - 12.76 ) | 8.66 ( 178.95 ) | 5.81 ( 4.22 ) | 2.54 ( 2.02 ) |
| Rash | 42 | 0.42 ( 0.31 - 0.57 ) | 0.42 ( 33.36 ) | 0.44 ( 0.34 ) | -1.2 ( -1.65 ) |
| Interstitial lung disease | 40 | 0.47 ( 0.34 - 0.64 ) | 0.47 ( 23.46 ) | 0.49 ( 0.37 ) | -1.04 ( -1.5 ) |
| Pulmonary embolism | 39 | 1.08 ( 0.78 - 1.5 ) | 1.08 ( 0.22 ) | 1.08 ( 0.82 ) | 0.11 ( -0.37 ) |
| Haemoptysis* | 37 | 1.51 ( 1.07 - 2.12 ) | 1.5 ( 5.69 ) | 1.46 ( 1.1 ) | 0.54 ( 0.05 ) |
| White blood cell count decreased | 34 | 1.18 ( 0.83 - 1.67 ) | 1.18 ( 0.84 ) | 1.16 ( 0.87 ) | 0.22 ( -0.29 ) |
| Dysphagia* | 34 | 1.64 ( 1.15 - 2.34 ) | 1.64 ( 7.6 ) | 1.57 ( 1.17 ) | 0.65 ( 0.14 ) |
| Infection* | 33 | 1.95 ( 1.36 - 2.81 ) | 1.95 ( 13.46 ) | 1.84 ( 1.35 ) | 0.88 ( 0.35 ) |
| Stomatitis | 32 | 1.16 ( 0.81 - 1.66 ) | 1.16 ( 0.65 ) | 1.15 ( 0.85 ) | 0.2 ( -0.32 ) |
| Hypomagnesaemia* | 32 | 3.88 ( 2.63 - 5.74 ) | 3.87 ( 53.96 ) | 3.27 ( 2.36 ) | 1.71 ( 1.16 ) |
| Respiratory failure | 31 | 0.69 ( 0.48 - 0.99 ) | 0.69 ( 4.16 ) | 0.7 ( 0.52 ) | -0.51 ( -1.03 ) |
| Hyponatraemia | 31 | 1.38 ( 0.95 - 1.99 ) | 1.37 ( 2.91 ) | 1.34 ( 0.99 ) | 0.43 ( -0.11 ) |
| Atrial fibrillation | 30 | 1.13 ( 0.78 - 1.64 ) | 1.13 ( 0.41 ) | 1.12 ( 0.82 ) | 0.16 ( -0.37 ) |
| Hepatitis* | 30 | 3.61 ( 2.42 - 5.39 ) | 3.6 ( 45.26 ) | 3.09 ( 2.21 ) | 1.63 ( 1.06 ) |
| Pruritus | 29 | 1.04 ( 0.71 - 1.52 ) | 1.04 ( 0.04 ) | 1.04 ( 0.76 ) | 0.05 ( -0.49 ) |
| Chest pain* | 28 | 1.71 ( 1.16 - 2.53 ) | 1.71 ( 7.37 ) | 1.63 ( 1.18 ) | 0.71 ( 0.14 ) |
| Neutrophil count decreased | 28 | 0.93 ( 0.63 - 1.36 ) | 0.93 ( 0.14 ) | 0.93 ( 0.68 ) | -0.1 ( -0.65 ) |
| Blood creatinine increased | 27 | 1.11 ( 0.75 - 1.64 ) | 1.11 ( 0.26 ) | 1.1 ( 0.79 ) | 0.14 ( -0.43 ) |
| Hypothyroidism | 27 | 1.23 ( 0.83 - 1.82 ) | 1.23 ( 1.05 ) | 1.21 ( 0.87 ) | 0.27 ( -0.29 ) |
| Renal failure | 26 | 1.18 ( 0.79 - 1.76 ) | 1.18 ( 0.68 ) | 1.17 ( 0.84 ) | 0.22 ( -0.35 ) |
| Epistaxis* | 26 | 1.84 ( 1.22 - 2.77 ) | 1.84 ( 8.85 ) | 1.74 ( 1.24 ) | 0.8 ( 0.22 ) |
| Neuropathy peripheral* | 25 | 1.81 ( 1.19 - 2.74 ) | 1.8 ( 7.97 ) | 1.71 ( 1.21 ) | 0.78 ( 0.18 ) |
| Septic shock* | 24 | 1.64 ( 1.08 - 2.51 ) | 1.64 ( 5.42 ) | 1.58 ( 1.11 ) | 0.66 ( 0.05 ) |

Abbreviation: Asterisks (*) indicate statistically significant signals in algorithm; ROR, reporting odds ratio; PRR, proportional reporting ratio; EBGM, empirical Bayesian geometric mean; EBGM05, the lower limit of the 95% CI of EBGM; IC, information component; IC025, the lower limit of the 95% CI of the IC; CI, confidence interval; PT, preferred term.

Supplementary Table 9:

Top 50 most frequent adverse events for Carboplatin excluding common medication co-usage at the PT level from FAERS data

| PT | Case numbers | ROR(95%Cl) | PRR(χ2) | EBGM(EBGM05) | IC(IC025) |
| --- | --- | --- | --- | --- | --- |
| Dyspnoea* | 56 | 2.06 ( 1.58 - 2.7 ) | 2.03 ( 29.33 ) | 2.02 ( 1.61 ) | 1.01 ( 0.62 ) |
| Dehydration* | 40 | 2.94 ( 2.14 - 4.03 ) | 2.9 ( 48.95 ) | 2.86 ( 2.19 ) | 1.51 ( 1.05 ) |
| Nausea | 39 | 1.36 ( 0.99 - 1.87 ) | 1.35 ( 3.55 ) | 1.35 ( 1.03 ) | 0.43 ( -0.03 ) |
| Diarrhoea | 33 | 0.72 ( 0.51 - 1.01 ) | 0.72 ( 3.65 ) | 0.72 ( 0.54 ) | -0.47 ( -0.97 ) |
| Anaemia* | 33 | 1.52 ( 1.07 - 2.15 ) | 1.51 ( 5.68 ) | 1.5 ( 1.13 ) | 0.59 ( 0.09 ) |
| Neutropenia* | 30 | 2.11 ( 1.47 - 3.03 ) | 2.09 ( 16.92 ) | 2.07 ( 1.53 ) | 1.05 ( 0.53 ) |
| Vomiting | 29 | 1.36 ( 0.94 - 1.96 ) | 1.35 ( 2.68 ) | 1.35 ( 0.99 ) | 0.43 ( -0.1 ) |
| Fatigue | 29 | 1.11 ( 0.77 - 1.61 ) | 1.11 ( 0.33 ) | 1.11 ( 0.82 ) | 0.15 ( -0.38 ) |
| Hypotension* | 28 | 4.3 ( 2.95 - 6.29 ) | 4.25 ( 67.76 ) | 4.15 ( 3.02 ) | 2.05 ( 1.51 ) |
| Febrile neutropenia* | 28 | 2.16 ( 1.48 - 3.15 ) | 2.15 ( 16.98 ) | 2.13 ( 1.55 ) | 1.09 ( 0.55 ) |
| Pneumonia | 26 | 0.93 ( 0.63 - 1.38 ) | 0.94 ( 0.12 ) | 0.94 ( 0.68 ) | -0.1 ( -0.66 ) |
| Thrombocytopenia* | 23 | 1.76 ( 1.16 - 2.66 ) | 1.75 ( 7.37 ) | 1.74 ( 1.23 ) | 0.8 ( 0.2 ) |
| Pyrexia | 20 | 0.84 ( 0.54 - 1.3 ) | 0.84 ( 0.62 ) | 0.84 ( 0.58 ) | -0.25 ( -0.89 ) |
| Pruritus* | 19 | 2.55 ( 1.61 - 4.02 ) | 2.53 ( 17.35 ) | 2.5 ( 1.71 ) | 1.32 ( 0.67 ) |
| Cough* | 19 | 1.76 ( 1.12 - 2.78 ) | 1.76 ( 6.15 ) | 1.75 ( 1.19 ) | 0.81 ( 0.15 ) |
| Pulmonary embolism | 18 | 1.55 ( 0.97 - 2.48 ) | 1.55 ( 3.48 ) | 1.54 ( 1.04 ) | 0.63 ( -0.04 ) |
| Platelet count decreased* | 18 | 1.9 ( 1.19 - 3.04 ) | 1.89 ( 7.53 ) | 1.88 ( 1.27 ) | 0.91 ( 0.24 ) |
| Chest pain* | 17 | 2.99 ( 1.85 - 4.85 ) | 2.98 ( 21.88 ) | 2.93 ( 1.96 ) | 1.55 ( 0.86 ) |
| White blood cell count decreased* | 16 | 2.1 ( 1.28 - 3.45 ) | 2.09 ( 9.01 ) | 2.07 ( 1.37 ) | 1.05 ( 0.34 ) |
| Pneumonitis | 16 | 0.92 ( 0.56 - 1.51 ) | 0.92 ( 0.11 ) | 0.92 ( 0.61 ) | -0.12 ( -0.82 ) |
| Dysphagia* | 16 | 2.72 ( 1.65 - 4.47 ) | 2.7 ( 16.89 ) | 2.67 ( 1.76 ) | 1.42 ( 0.71 ) |
| Leukopenia* | 15 | 2.21 ( 1.33 - 3.7 ) | 2.21 ( 9.75 ) | 2.19 ( 1.42 ) | 1.13 ( 0.4 ) |
| Hypokalaemia* | 15 | 2.95 ( 1.77 - 4.93 ) | 2.94 ( 18.78 ) | 2.89 ( 1.88 ) | 1.53 ( 0.8 ) |
| Oesophagitis* | 15 | 4.88 ( 2.91 - 8.18 ) | 4.85 ( 44.21 ) | 4.71 ( 3.05 ) | 2.24 ( 1.5 ) |
| Respiratory failure | 14 | 1.23 ( 0.73 - 2.09 ) | 1.23 ( 0.6 ) | 1.23 ( 0.79 ) | 0.3 ( -0.45 ) |
| Haemoglobin decreased* | 14 | 2.42 ( 1.42 - 4.11 ) | 2.41 ( 11.33 ) | 2.38 ( 1.53 ) | 1.25 ( 0.5 ) |
| Atrial fibrillation* | 13 | 2.43 ( 1.4 - 4.22 ) | 2.43 ( 10.72 ) | 2.4 ( 1.51 ) | 1.26 ( 0.48 ) |
| Flushing* | 13 | 9.8 ( 5.57 - 17.25 ) | 9.74 ( 95 ) | 9.14 ( 5.69 ) | 3.19 ( 2.39 ) |
| Asthenia | 13 | 0.73 ( 0.42 - 1.27 ) | 0.73 ( 1.26 ) | 0.74 ( 0.47 ) | -0.44 ( -1.22 ) |
| General physical health deterioration | 12 | 1.03 ( 0.58 - 1.81 ) | 1.03 ( 0.01 ) | 1.03 ( 0.64 ) | 0.04 ( -0.77 ) |
| Infection* | 12 | 2.2 ( 1.24 - 3.89 ) | 2.19 ( 7.64 ) | 2.17 ( 1.34 ) | 1.12 ( 0.31 ) |
| Abdominal pain | 12 | 1.6 ( 0.91 - 2.84 ) | 1.6 ( 2.68 ) | 1.59 ( 0.99 ) | 0.67 ( -0.14 ) |
| Acute kidney injury | 12 | 1.48 ( 0.84 - 2.63 ) | 1.48 ( 1.86 ) | 1.48 ( 0.91 ) | 0.56 ( -0.25 ) |
| Immune-mediated enterocolitis* | 12 | 4.9 ( 2.75 - 8.73 ) | 4.87 ( 35.65 ) | 4.73 ( 2.92 ) | 2.24 ( 1.42 ) |
| Haemoptysis | 11 | 1.36 ( 0.75 - 2.47 ) | 1.36 ( 1.05 ) | 1.36 ( 0.82 ) | 0.44 ( -0.4 ) |
| Immune-mediated hypothyroidism* | 11 | 16.67 ( 8.89 - 31.26 ) | 16.58 ( 143.03 ) | 14.83 ( 8.76 ) | 3.89 ( 3.01 ) |
| Neutrophil count decreased | 11 | 1.56 ( 0.86 - 2.83 ) | 1.56 ( 2.17 ) | 1.55 ( 0.94 ) | 0.63 ( -0.21 ) |
| Decreased appetite | 10 | 0.5 ( 0.27 - 0.93 ) | 0.5 ( 4.92 ) | 0.51 ( 0.3 ) | -0.98 ( -1.86 ) |
| Heart rate increased* | 10 | 6.15 ( 3.26 - 11.62 ) | 6.13 ( 41.03 ) | 5.9 ( 3.47 ) | 2.56 ( 1.67 ) |
| Interstitial lung disease | 10 | 0.53 ( 0.28 - 0.98 ) | 0.53 ( 4.2 ) | 0.53 ( 0.32 ) | -0.91 ( -1.79 ) |
| Anaphylactic reaction* | 10 | 6.97 ( 3.68 - 13.19 ) | 6.94 ( 48.31 ) | 6.64 ( 3.89 ) | 2.73 ( 1.84 ) |
| Erythema* | 10 | 2.85 ( 1.52 - 5.34 ) | 2.84 ( 11.68 ) | 2.8 ( 1.66 ) | 1.49 ( 0.6 ) |
| Myocardial infarction* | 10 | 2.75 ( 1.47 - 5.16 ) | 2.74 ( 10.87 ) | 2.71 ( 1.6 ) | 1.44 ( 0.56 ) |
| Infusion related reaction* | 10 | 2.78 ( 1.48 - 5.21 ) | 2.77 ( 11.12 ) | 2.74 ( 1.62 ) | 1.45 ( 0.57 ) |
| Constipation | 10 | 0.91 ( 0.49 - 1.69 ) | 0.91 ( 0.1 ) | 0.91 ( 0.54 ) | -0.14 ( -1.02 ) |
| Hypoxia* | 9 | 2.02 ( 1.05 - 3.91 ) | 2.02 ( 4.56 ) | 2 ( 1.15 ) | 1 ( 0.08 ) |
| Rash | 9 | 0.31 ( 0.16 - 0.6 ) | 0.31 ( 13.63 ) | 0.32 ( 0.18 ) | -1.66 ( -2.58 ) |
| Weight decreased | 9 | 0.97 ( 0.51 - 1.88 ) | 0.97 ( 0.01 ) | 0.97 ( 0.56 ) | -0.04 ( -0.96 ) |
| Neutropenic sepsis* | 9 | 4.25 ( 2.18 - 8.26 ) | 4.23 ( 21.53 ) | 4.13 ( 2.37 ) | 2.05 ( 1.12 ) |
| Deep vein thrombosis | 9 | 1.92 ( 0.99 - 3.71 ) | 1.92 ( 3.89 ) | 1.9 ( 1.1 ) | 0.93 ( 0.01 ) |

Abbreviation: Asterisks (*) indicate statistically significant signals in algorithm; ROR, reporting odds ratio; PRR, proportional reporting ratio; EBGM, empirical Bayesian geometric mean; EBGM05, the lower limit of the 95% CI of EBGM; IC, information component; IC025, the lower limit of the 95% CI of the IC; CI, confidence interval; PT, preferred term.
